# Supplementary material for: CAT Bridge: an efficient toolkit for gene–metabolite association mining from multiomics data
Source: Gigascience. 2024 Nov 8;13:giae083. doi: 10.1093/gigascience/giae083 (PMC11548955; doi:10.1093/gigascience/giae083)
Supplement: giae083_GIGA-D-24-00083_Revision_1 [file giae083_giga-d-24-00083_revision_1.pdf]

GigaScience

CAT Bridge: An Efficient Toolkit for Gene-Metabolite Association Mining from Multi-Omics Data

--Manuscript Draft--

**Manuscript Number:**	GIGA-D-24-00083R1
**Full Title:**	CAT Bridge: An Efficient Toolkit for Gene-Metabolite Association Mining from Multi-Omics Data
**Article Type:**	Technical Note
**Funding Information:**	Key Technology Research and Development Program of Shandong Province (ZR202211070163) — Dr. Li Guo<br>Natural Science Foundation of Shandong Province (ZR2023JQ010) — Dr. Li Guo<br>Taishan Scholar Foundation of Shandong Province — Dr. Li Guo
**Abstract:**	Background: With advancements in sequencing and mass spectrometry technologies, multi-omics data can now be easily acquired for understanding complex biological systems. Nevertheless, substantial challenges remain in determining the association between gene-metabolite pairs due to the non-linear and multifactorial interactions within cellular networks. The complexity arises from the interplay of multiple genes and metabolites, often involving feedback loops and time-dependent regulatory mechanisms that are not easily captured by traditional analysis methods.<br>Findings: Here, we introduce Compounds And Transcripts Bridge (abbreviated as CAT Bridge, available at https://catbridge.work), a free user-friendly platform for longitudinal multi-omics analysis to efficiently identify transcripts associated with metabolites using time-series omics data. To evaluate the association of gene-metabolite pairs, CAT Bridge is a pioneering work benchmarking a set of statistical methods spanning causality estimation and correlation coefficient calculation for multi-omics analysis. Additionally, CAT Bridge features an artificial intelligence (AI) agent to assist users interpreting the association results.<br>Conclusions: We applied CAT Bridge to experimentally obtained Capsicum chinense (chili pepper) and public human and Escherichia coli (E. coli) time-series transcriptome and metabolome datasets. CAT Bridge successfully identified genes involved in the biosynthesis of capsaicin in C. chinense. Furthermore, case study results showed that the convergent cross mapping (CCM) method outperforms traditional approaches in longitudinal multi-omics analyses. CAT Bridge simplifies access to various established methods for longitudinal multi-omics analysis, and enables researchers to swiftly identify associated gene-metabolite pairs for further validation.
**Corresponding Author:**	Li Guo<br>Peking University Institute of Advanced Agricultural Sciences<br>Weifang, CHINA
**Corresponding Author Secondary Information:**	
**Corresponding Author's Institution:**	Peking University Institute of Advanced Agricultural Sciences
**Corresponding Author's Secondary Institution:**	
**First Author:**	Bowen Yang
**First Author Secondary Information:**	
**Order of Authors:**	Bowen Yang<br>Tan Meng<br>Xinrui Wang

Powered by Editorial Manager® and ProduXion Manager® from Aries Systems Corporation

	Jun Li
	Shuang Zhao
	Yingheng Wang
	Shu Yi
	Yi Zhou
	Yi Zhang
	Liang Li
	Li Guo
**Order of Authors Secondary Information:**	
**Response to Reviewers:**	Dear Editor,<br><br>Enclosed is our revised manuscript entitled "CAT Bridge: An Efficient Toolkit for Gene-Metabolite Association Mining from Multi-Omics Data" that we would like to submit again as a Technical Note to GigaScience for consideration.<br><br>We are very grateful to you and the reviewers for giving us to valuable comments and suggestions to help improve our manuscript. We have been working on revising the manuscript carefully according to these comments and questions. Particularly we have addressed the reviewer concern about the LLM-based functionality and your requests on the RRID identifiers, which are described in our point-to-point response to all comments along with the revised manuscript. We trust that the revisions have substantially improved the manuscript, making it more suitable for publication in GigaScience.<br><br>Please let me know if further information is needed regarding the submission. We are looking forward to hearing from you.<br><br>Yours sincerely,<br><br>Li Guo, Ph.D.<br>Principal Investigator<br>Peking University Institute of Advanced Agricultural Sciences<br>Weifang, Shandong, China<br>li.guo@pku-iaas.edu.cn<br><br>Response to editor comments:<br>I'd like to emphasize that I completely share the major concerns of reviewer #1 regarding the use of an LLM for interpreting the data. The validity and factual correctness of this approach needs to be backed up with hard evidence, and limitations and caveats properly addressed. If a convincing validation of the LLM-based functionality is not possible, it might be preferable to omit or replace the GTP integration.<br>Author response: Thank you for your suggestion. We have thoroughly discussed this issue in our response to Reviewer #1. We have added a description of the method and on how to reduce AI hallucination problems by using appropriate prompt words and a low-temperature setting; we have also included a warning about the risks of using this feature. If the responses generated by the Large Language Model (LLM) are still considered misleading, we are prepared to completely remove this feature from CAT Bridge until the techniques are more mature.<br><br>In addition, please register any new software application in the bio.tools and SciCrunch.org databases to receive RRID (Research Resource Identification Initiative ID) and biotoolsID identifiers, and include these in your manuscript.<br>Author response: We have submitted CAT Bridge to receive the Research Resource Identification Initiative ID (SCR_025410) and biotoolsID (cat_bridge). And this information has been included in the Availability of source code and requirements section of our manuscript.<br><br>Response to reviewer comments:

Powered by Editorial Manager® and ProduXion Manager® from Aries Systems Corporation

Reviewer #1:
The authors introduce a useful tool (CAT Bridge) for integrating multiple causal and correlative analyses for multi-omics integration, which also includes a visualization and LLM component. The authors further provide two case studies (human and plant) illustrating the utility of CAT Bridge. I believe that this work should be published, as it contributes to the field of multi-omics analysis.
Author response: We are truly grateful for your positive acknowledgment of our work on the CAT Bridge tool. We believe CAT Bridge will serve as a beneficial resource for researchers in their analytical endeavors.
However, I am very concerned about the lack of description regarding the LLM. As explained by Mittelstadt et al (https://www.nature.com/articles/s41562-023-01744-0), LLMs do not always provide factual answers. The authors need to justify the use of the LLM to determine the relevance of a gene-metabolite association. In particular, the authors should add to the main text (or at least the supplementary) a detailed description of the prompt construction and should justify why this prompt is expected to result in factual information. Furthermore, the authors should discuss the caveats of using LLMs in this context, starting with the linked article above. I believe that the manuscript will only be publishable once this concern is addressed.
Author response: We are grateful for the reference provided, Mittelstadt et al. have thoroughly discussed the issue of LLMs regarding their potential to generate misleading information in the reference, and we acknowledge that current LLMs might not be absolutely trustworthy. However, we believe that the integration of an AI Agent is a valuable feature with acceptable limitations, especially for computational biologists who may lack domain knowledge of the molecular mechanisms underlying specific biological questions. Researchers can therefore prioritize the genes suggested by the LLM for downstream research and validation. As an additional layer of information to inspire further investigation, LLMs are not meant to replace empirical validation but to provide a starting point for experimental validation based on the synthesis of prior knowledge. Users of CAT Bridge can decide if they would like assistance from the AI agent, or trust their interpretation of the output from CAT Bridge.
To minimize the potential misguidance caused by hallucinations in the Master of Laws, we have set reasonable context settings and prompts, as well as a more conservative temperature setting (which will be elaborated on in the following content). We have also added additional disclaimers to the responses generated by the artificial intelligence, these will inform researchers that the results from the AI Agent are derived from an LLM's output, which does not ensure factual accuracy, and that manual verification is essential. Furthermore, this feature requires the user to obtain an OpenAI API key which is not provided in the web server version. We trust that users who engage with this feature generally possess a robust understanding of the operational mechanisms and inherent characteristics of LLMs' outputs.
In addition, we have added a new section detailing the LLM's methodology to the revised supplementary text: “The artificial intelligence (AI) Agent was developed utilizing the OpenAI API to integrate large language model (LLM) GPT-3.5 turbo. The prompts of the AI agent are constructed based on the top 100 genes ranked from heuristic ranking, combining the genes and their functional annotations to create a list of formatted strings, followed by a question regarding their potential involvement in the synthesis of the target metabolite (Which one may be involved in the synthesis of [target metabolite]). To mitigate the risk of hallucinations or factual inaccuracies in the LLM's response, the ‘system' role message contextualizes the AI as a knowledgeable biological chemist. Additionally, the temperature setting was lowered to 0.2, which resulted in more cautious and deterministic outputs. This approach minimizes the chances of deviating from factual data, favoring factual accuracy over creative but potentially speculative responses” (Lines 74-84).
We also incorporated a discourse on the caveats of utilizing LLMs into the Discussion: “LLM-based AI agents have shown a wide range of applications in various fields, but the hallucination of knowledge deficiency remains an issue that can mislead users. Although we have enhanced the credibility of the AI agent response via appropriate prompt and low temperature, this cannot be regarded as a substitute for professional knowledge and experimental verification, but merely serves as a starting point for verification, the definitive conclusions still require manual assessment by researchers” (Lines 387-392).
Finally, we also added this statement to the end of the AI agent response: “This response is generated by a Large Language Model (GPT 3.5 turbo) and should not be considered as a substitute for professional judgment. Users are advised to verify the

Powered by Editorial Manager® and ProduXion Manager® from Aries Systems Corporation

accuracy of the information through rigorous literature review and, where necessary, experimental confirmation.”
If the responses generated by the LLM are still regarded as misleading, we can completely remove this feature from CAT Bridge until the techniques mature.

In addition, the authors are recommended to address the following more minor concerns:
Implementation:
Your "example file" links at https://catbridge.work are broken. Please fix this.
Author response: We apologize for the inconvenience caused by the broken links, we have addressed the issue, and the links are now functional.

Abstract:
1.Line 32: "Nevertheless, substantial challenges remain in determining the association between gene-metabolite pairs due to the complexity of cellular networks." This is not a clear statement. What about the complexity of cellular networks presents challenges in determining the associations?
Author response: We appreciate the opportunity to clarify the statement regarding the challenges presented by the complexity of cellular networks in determining gene-metabolite associations. The complexity arises due to several factors:
●Non-linear Relationships: Gene expression and metabolite production often exhibit non-linear dynamics, complex regulatory networks, and feedback loops, making it difficult to predict based on simple correlation-based models.
●Non-one-to-one Correspondence: Multiple genes can influence a single metabolite, and conversely, a single gene can affect multiple metabolites. This complexity makes it challenging to establish direct associations.
●Temporal Dynamics: The temporal dynamics in cellular networks pose challenges, as metabolite levels often lag behind changes in gene expression. This inherent delay complicates the direct determination of their association.
Therefore, we have revised the manuscript text accordingly: “Nevertheless, substantial challenges remain in determining the association between gene-metabolite pairs due to the non-linear and multifactorial interactions within cellular networks. The complexity arises from the interplay of multiple genes and metabolites, often involving feedback loops and time-dependent regulatory mechanisms that are not easily captured by traditional analysis methods” (Lines 32-36).

2.Make sure you are using present tense consistently, not past tense (Line 39).
Author response: Thank you for the suggestion. We have revised the manuscript text to ensure consistent use of the present tense.

3.Please use the scientific name with the common name in parentheses as follows: Capsicum chinense (chili pepper). Use only the scientific name throughout the rest of the document (Line 41).
Author response: Thanks for your suggestion. We have revised the manuscript to comply with the requested format for scientific names. The species is now initially presented as Capsicum chinense (chili pepper), and only the scientific name is used throughout the remainder of the manuscript.

Background:
1.Line 56: "Background" should not be plural.
Author response: Revised.

2.Lines 59-60: More comprehensive than what? Please elaborate here.
Author response: Thanks for the questions. We meant to say that it is more comprehensive than single-omic studies. The term "comprehensive" refers to the integration of multiple layers of molecular information and inter-level regulatory relationships. This integration enables us to have a broader picture of the biological system, thus determining whether observed changes at a molecular level from broader alterations across a metabolic pathway, or only regarding a specific molecule. Therefore, it provides a more systematic understanding of the biological mechanism, making it more comprehensive than single-omic studies.
We revised the manuscript as follows: "multi-omics data analysis is crucial for understanding intricate biological mechanisms from a more comprehensive perspective than single omics, this holistic analysis allows us to explore the interplay

between different molecular levels.” (Lines 59-62).

3.In Line 60, please include and familiarize yourself with the following reference: Eicher, T., G. Kinnebrew, A. Patt, K. Spencer, K. Ying, Q. Ma, R. Machiraju and E. A. Mathé (2020). "Metabolomics and Multi-Omics Integration: A Survey of Computational Methods and Resources." Metabolites 10: 202.
Author response: Thank you for recommending this review paper. We have carefully reviewed the literature and cited it, as it provides a comprehensive survey of computational methods and resources for metabolomics and multi-omics integration. It specifically discusses time series studies in omics, which is the main focus of our paper. This reference emphasizes the importance of addressing the lag issues between different omics layers. It uses Dynamic Time Warping (DTW), which is also integrated into CAT Bridge, as an example to illustrate how to align time points. Such specific data types are often overlooked in the research community's reviews (Lines 62).

4.Lines 67-68: Citation needed.
Author response: We have now included the necessary citations (doi: 10.1016/j.csbj.2021.09.001) in Lines 69-70 to support our statements, ensuring that our claims are well-substantiated.

5.Line 72: Please use the scientific name with the common name in parentheses.
Author response: Thank you for the reminder, we have updated our manuscript, now listing both the scientific and common names for two species: Solanum lycopersicum (tomato) and Oryza sativa (rice) (Line 74).

6.Lines 74-77: Citations needed.
Author response: We have now included the necessary citation (doi.org/10.1039/D2NP00032F and doi:10.3390/metabo10050202) in lines 77-80 to support this statement.

7.Lines 77-78: Give an example of biologically naïve conclusions from purely data-driven strategies.
Author response: Thanks for your suggestion! To help users understand the concept, we have included a classic example in our manuscript that shows how biologically naïve conclusions came from purely data-driven strategies. The manuscript has been revised as follows: “For example, a purely data-driven approach might incorrectly link low cholesterol levels with higher mortality rates, suggesting that lower cholesterol is detrimental. However, the actual cause is underlying diseases like cancer, which cause both low cholesterol and increased mortality” (Lines 83-86).

8.Line 78: Discuss how the machine learning models could address the drawbacks of the correlation models and vice-versa.
Author response: We apologize for the ambiguity. The "two methodologies" mentioned here refers to data-driven and knowledge-driven methods. To avoid ambiguity, we revised the sentence as "Therefore, integrating data-driven and knowledge-driven methodologies may offer a more comprehensive and accurate interpretation of multi-omics data. (Lines 87-88)"
The correlation models identify new connections and provide quantitative analysis, while knowledge-driven methods avoid many false positives caused by coincidental similarities to true functional gene expression patterns in situations where data is scarce. Thus, this combination improves generalizability and increases the interpretability of the model.

Materials and Methods:
1.It seems that CAT Bridge needs to be run on one metabolite at a time. In this case, I would not use the term "gene-metabolite pair association" in Line 104, but rather "associations between genes and the target metabolite".
Author response: Thank you for your suggestion, we have made the change in our manuscript (Lines 112-113). This revision enhances the clarity of our presentation and avoids any potential misunderstanding regarding the scope of CAT Bridge.

2.Line 115: Clearly state which of these methods are non-linear and which address the lag issue.

Powered by Editorial Manager® and ProduXion Manager® from Aries Systems Corporation

Author response: We have provided a detailed explanation of the methods in the revised manuscript (Table 1). Convergent Cross Mapping (CCM) and Dynamic Time Warping (DTW) are non-linear methods. CCM reconstructs the state space to detect causality in non-linear systems, while DTW allows flexible matching to handle non-linear alignments of time series data. Granger Causality (Granger) and Canonical Correlation Analysis (CCA) are inherently linear methods, although there are extensions such as non-linear Granger and kernel CCA that can address non-linear relationships. For handling time lag, CCM, Granger Causality, DTW, and the Cross-Correlation Function (CCF) are capable of addressing this issue. CCM and DTW inherently consider time lag through state space reconstruction and optimal matching, respectively. Granger Causality explicitly incorporates lagged values in its regression model, and CCF measures correlation at various time lags. Although standard CCA does not handle time lags, time-lagged variations of CCA can address this problem.

3.Line 136: Your figures are out of order (Figure 1B comes after Figure 2B).
Author response: Thanks for your comment. We apologize for the mistake. We have revised the manuscript to give the correct figure reference. The previous mention of "Figure 1B" has been revised to "Figure 2B”.

4.Please take a look at the Minimum Standards Reporting Checklist (https://academic.oup.com/gigascience/pages/Minimum_Standards_of_Reporting_Checklist). In particular:
a. In the section starting at Line 153, list the number of seedlings used. b. Were all timepoints collected from all seedlings? List the total number of samples. c. How many mg were collected per sample (can use a range here)? d. 3 biological replicates per seedling? Give more detail here. e. What machine was used for the ultrasonic process? If frequency settings are permitted by the machine, list the settings used. f. How many of the 28 younger and 54 older adults had both transcriptome and metabolome data?
Author response: Thanks for your suggestion, we have revised our manuscript by considering this standard:
Line 167 to 188:
“To test the effectiveness of CAT Bridge across different species, especially its applicability to non-model organisms, we collected transcriptome sequencing and metabolic profiling data from Capsicum chinense, focusing on one of its trademark natural products, capsaicin. C. chinense seedlings were divided into three groups, each containing 15 seedlings, grown in a greenhouse of Peking University Institute of Advanced Agricultural Sciences with a controlled environment of 25°C temperature, a light-dark cycle of 16 hours light and 8 hours dark, and 70% relative humidity. The fruits of C. chinense were sampled at seven distinct time points, starting from the day of flowering, i.e. 0 day post-anthesis (DPA) during which flowers were collected, followed by fruit harvest on days 7, 16, 30, 50, 55, and 60 DPA. For each time point, we sampled 15 fruits from each group of seedlings, yielding a total of 45 sampling per time point. For each group, we utilized 1.0 mL of 70% aqueous methanol per sample, with a sample weight of approximately 50mg (aside from the 7 DPA samples which averaged 27.3mg). The samples were ground and freeze-dried in liquid nitrogen. Each sample was then extracted using 1.0 mL of 70% aqueous methanol for every 50 mg of sample. Following extraction, the samples underwent ultrasonic treatment using an Ultrasonic Cell Disruptor SCIENTZ-IID (Ningbo Scientz Biotechnology Co., LTD., China) at a frequency of 40 kHz for 10 minutes at room temperature. Standards were prepared as follows: a mixed standard solution, ranging from 20-50 μg/mL, was prepared using MS-grade methanol. For the amino acid standard solution, a 1 mg/mL stock solution was initially prepared in water, and then diluted with 50% methanol to achieve a final concentration of 50 μg/mL. Three biological sample replicates were utilized in the subsequent transcriptome and metabolome analyses.”
Lines 226 - 229:
“The data was sourced from a published aging study that sampled transcriptome and metabolome data from 28 younger (20 to 25 years) and 54 older (55 to 66 years) female human donors. 13 time points are included in them where both the transcriptome and the metabolome data were detected.”

5.Line 209: "Younger" and "older" are better terms.
Author response: We appreciate your suggestion. These terms have been updated throughout the manuscript to ensure consistency and precision in age group

Powered by Editorial Manager® and ProduXion Manager® from Aries Systems Corporation

descriptions.

Results:
1.Line 248: How does the AI agent analyze the functional annotations?
Author response: Thanks for the question. The specific description of the AI agent is discussed in our response to your previous comment above. Specifically, for this case study, the top 100 genes in heuristic ranking ranked by the CCM-based method along with their functional annotations (generated by eggNOG-mapper) were fed to a GPT 3.5-based LLM. The context was set as a professional biochemist, with the inquiry focusing on which gene might be involved in the synthesis of capsaicin. The AI agent successfully located BC332_05016, a gene encoding Acyl-transferase, a key enzyme of capsaicin biosynthesis.

2.Lines 281-282: "This illustrates the advantage of causal relationship modeling methods over traditional methods".
Author response: Thanks for your correction, we have revised the manuscript accordingly (Line 308).

3.Line 290: Please also include the updated IntLIM paper (IntLIM 2.0): Eicher, T., K. D. Spencer, J. K. Siddiqui, R. Machiraju and E. A. Mathe (2023). "IntLIM 2.0: identifying multi-omic relationships dependent on discrete or continuous phenotypic measurements." Bioinformatics Advances 3(1): vbad009.
Author response: Thank you for your suggestion on the citation. We have included this IntLIM2.0 paper to the revised manuscript (Line 339).

4.Make sure the colors are consistent in Table 1.
Author response: We have revised the table to ensure that the colors used are consistent.

5.Line 156: The scientific name of the pepper species is inconsistent with other areas of the text.
Author response: Thank you for your reminder, we have corrected the scientific name.

Figures:
1.S1 should be provided as a table, not a figure.
Author response: Thanks for your suggestion! This figure was used to show the visual output of CAT Bridge and contained content already shown in the supplementary table. Thus, we have removed Figure S1.

2.Please make S2 larger. It is difficult to read.

Author response: Revised as suggested.

3.S3 needs labels (x axis, y axis, legend).
Author response: Revised as suggested.
Thank you once again for your meticulous review of our work, especially for the detailed discussion regarding the LLMs and your attention to the scientific details within our manuscript, ensuring that it is presented with clarity, accuracy, and integrity.

Reviewer #2:
To the authors,
Thank you for the opportunity to review the manuscript GIGA-D-24-00083. The authors created a tool to predict association between genes and metabolites using various algorithms. The authors provide the tool as a web application, and as a python package. To get the reciprocal relationship between gene and metabolites, i.e. which metabolites can change which gene or vice versa, this tool can be a toolkit for the biologist or bioinformatician.
The tool has application specially the relationship between changes in genes and metabolites is not direct, many complex mechanisms exist e.g. epigenetic or polymorphism. So the tool can be alternate to other available tools.
Also, the manuscript brings the community focus on causal relationships instead of just correlation based relationships. The tool used temporal causality algorithms for predicting relationships between genes and metabolites.
Author response: Thank you for your positive comments, highlighting temporal

causality between omics data and providing democratized analytical software were key objectives of our work.

However, I recommend major revisions before publication. Here are my reasons and comments for the revisions:
General issues with web accessibility and package installation :
1.There are concerns about web accessibility, as indicated by web browsers flagging the connection as insecure. This may stem from geographical restrictions or the absence of HTTPS certification. Addressing these issues would ensure secure access to the server.
Author response: Thanks for your excellent suggestion and for bringing up this issue. Currently, more and more browsers enforce stringent security protocols. Therefore, we have transitioned to using HTTPS to ensure a more secure protocol for accessing the server.

2.Despite successful initiation of the client application from the git repository as a python module, no results were generated upon launching. It is suggested that the authors distribute the tool as a Docker image to facilitate seamless usage, eliminating concerns regarding dependencies and version compatibility.
Author response: Thank you for the feedback and suggestion regarding the client application. We have created and distributed a Docker image, and it's currently accessible on Docker Hub (https://hub.docker.com/r/bowen172/cat-bridge). Additionally, we have provided complete environment information (https://github.com/Bowen999/CAT-Bridge/tree/main/client), enabling users to recreate the same environment using conda. This method is more straightforward and can avoid issues related to hardware architecture differences. By providing two files, it can avoid issues with version compatibility and environment setup.

Other comments:
1.There are inconsistencies regarding data preprocessing. While the manuscript mentions that the tool will handle preprocessing, it also indicates that users need to provide processed files. Clarification is needed on whether preprocessing is required. It seems, the tool required preprocessed data.
Author response: Thanks for your comment. The software indeed requires preprocessed gene expression matrices and metabolite concentration matrices, instead of raw data. The preprocessing mentioned in the manuscript refers to handling missing values, standardization, and other such adjustments within these matrices. We have revised the manuscript to replace preprocessing" with "data processing" for clarity.

2.For clarity use "causality and correlation" instead of "causality/correlation" algorithms.
Author response: We agree with you that this is a clearer terminology and avoids any ambiguity that might arise from the use of a slash. We have updated the manuscript to reflect this change. Thank you for the suggestion.

3.Can the tool process any new temporal numerical data series, or does it specifically filter for genes? For instance, if I provide a list of proteins along with a list of genes, will I receive the association between them? It is suggested to include this in the discussion section.
Author response: We appreciate your insightful question regarding the tool's capacity to process various temporal numerical data series.
Our paper primarily focuses on the discovery of associations between genes and metabolites. However, the algorithms integrated into CAT Bridge were originally applied in other fields. For example, Convergent Cross Mapping (CCM), the highest-performing algorithm in this study, was initially used in the ecological study. Similarly, the Granger causality test originated in an economics study. Theoretically, these algorithms have the potential to analyze time-series data of other molecular types, such as the associations between genes and proteins, proteins, and metabolites (and vice versa). To demonstrate the tool's versatility beyond gene-metabolite pairs, we have included a case study (Case Study 3) that investigates the relationship between metabolites and genes (more particularly, transcription factors). For further information, see our response to question 9 in this letter.
We have also added the following content to the Discussion regarding this issue: “We tested the performance of CAT Bridge on the gene-to-metabolite and metabolite-to-

gene tasks in this research, since CAT Bridge is derived by integrating algorithms originating from different fields and its theoretical basis suggests it has generalizability, we believe that it can be used for investigating associations between other different molecular levels (such as gene to protein). Nonetheless, validating its performance across these different interactions is necessary for the conclusion due to the variations in complexity and regulatory timing at different molecular levels (Lines 380-384).”

4.Does the tool offer the capability to generate a causal diagram or network from these vectors, thereby providing visual support for their assertion regarding the causal relationship between metabolites and genes? If the author is working in this direction, it is suggested that information can be added in the discussion section.
Author response: Thanks for your question. Yes, we are actively exploring methods to visualize biological causal networks, including illustrating the time lag between metabolite concentrations and gene expression patterns, and integrating KEGG metabolic pathways information to construct molecular networks. We plan to include this functionality in our future work.
We have added the following statement to the Discussion: "Future work will involve determining response times between molecules at different levels, visualizing these lagged non-linear relationships, and constructing molecular networks based on temporal causality and metabolic pathways (Lines 400-402).

5.What definition of causal relationship did the author use, and could they provide a citation for their definition. Predictability or any other criteria were used for causal relationships. Please include the definition or criteria in the introduction and method section.
Author response: Thanks for your suggestion. The definition of causality utilized in this manuscript aligns with the concept of predictability, whereby causality is inferred based on the ability of one time series (gene expression) to predict another (metabolite concentration). This inference is implemented through the application of Convergent Cross Mapping and Granger Causality test.
To improve clarity, additional information was incorporated into the Background section with citations: "A more reliable solution is to use causality, which is inferred based on the ability of one time series (e.g., gene expression) to predict another (e.g., metabolite concentration), to replace the correlation coefficient (Lines 80-82)."
Similarly, the Methods section has been revised to include detailed descriptions: "including Granger Causality (Granger) and Convergent Cross Mapping (CCM) by evaluating the predictability of metabolite concentration from gene expression to represent causality (Lines 126-127)."

6.What are the minimum or maximum time points (interval) for input files? e.g. will the tool work if I provide only two times points or If I provide 48 times points. Please include the information in the method section.
Author response: Good question! In general, an increased number of sampling time points contributes to greater reliability of the results, as this mitigates the issue of false positive candidate genes that exhibit similar expression patterns to truly functional genes by coincidence. And in cases where time points are numerous, particularly with intensive sampling at short intervals, it is crucial to select a reasonable time lag. The example involving E. coli, as discussed in response to Question 9, effectively demonstrates this point.

7.What is the influence of the number of time points on the vector relationship presented in the paper? Have any studies by the authors addressed this question? Please include the results and discussion.
Author response: In our study, the influence of the number of time points primarily arises from the sampling interval, which affects the lag time settings, and whether the number of time points is sufficient to achieve comprehensive causal modeling results. The detail about this topic is discussed in Question 6 and Question 9.

8.Could the authors clarify which heuristic algorithm was employed for ranking the genes? Additionally, can they elaborate on how their approach to gene ranking is heuristic rather than relying on mathematical optimization or algorithmic methods? Clarification on the term "heuristic" would be beneficial.
Author response: The term "heuristic" refers to problem-solving techniques that employ practical methods to find solutions when classic methods are impractical. In our case,

we have utilized a heuristic approach to rank genes based on their association with metabolites, rather than employing strict mathematical methods. The heuristic algorithm we employed for gene ranking integrates multiple factors, including the strength of the association between genes and metabolites as measured by various statistical methods, fold change values, and, optionally, gene function annotations. This composite scoring system is designed to prioritize genes that are more likely to be biologically relevant with the target gene. This ranking is not the result of a single, universally optimal solution but rather a practical method to prioritize candidate genes for further investigation, this approach is flexible and can be adapted based on the availability of data and specific research questions.
We did not adopt strict mathematical methods but rather heuristic ranking for the following reasons:
●Complexity of Biological Systems: Biological systems are inherently complex, with many variables and interactions that are not fully captured by strict mathematical models, especially for models applicable to different species. Heuristic methods allow for flexibility in handling this complexity.
●Data Characteristics: The data from omics studies can be noisy and high-dimensional, often with missing values or incomplete information. Heuristic approaches can be more robust to these data characteristics compared to strict mathematical methods that may require complete and clean data.
●Scalability and Adaptability: Heuristic approaches are generally more adaptable to different research questions and can be scaled to various sizes of datasets without significant modifications.

9.Could the authors offer an example from studies conducted on yeast, E. coli, or other simple organisms, demonstrating how changes in gene sequences have readily been observed to affect metabolite levels? Please include that in the results section.
Author response: Thank you for the suggestion. Yes, in simple organisms, the regulation relationship between genes and metabolites also exhibits time lag and non-linear characteristics. Previous researchers have observed these phenomena in E. coli networks (https://doi.org/10.1089%2Fomi.2010.0074). To validate this and test CAT Bridge's performance on this task, we utilized an open-sourced transcriptomics and metabolomics dataset from E. coli (https://doi.org/10.1038/s41467-019-12474-1) to perform a new case study, which we have included as a new results section in the manuscript.
We have updated the Materials and Methods and Results section of our manuscript to include the following example:
Lines 232 to 237:
"The dataset for E. coli originated from a study on the interactions between metabolites and genes within the bacterium [33]. Researchers manipulated the culture conditions of E. coli, alternating between growth and starvation phases. They then collected the transcriptome and metabolome under these varying conditions to systematically explore how metabolites influence transcription factors. The dataset includes 29 time points where both transcriptomic and metabolomic information were concurrently obtained."
Lines 311 to 331:
“In the third study, we utilized a transcriptomic and metabolomic dataset from E. coli to demonstrate the capabilities of CAT Bridge in analyzing simple organisms. The dataset contains gene expression and metabolite concentration data across 29 time points, and it was originally generated by Lempp et al. to investigate the allosteric regulation of transcription factors (TFs) by metabolites. They constructed a "literature network" of known metabolite-TF interactions, including 16 interactions as activations of the TF by metabolites. Using Kinetic Correlation, which accommodates time lags, Lempp et al. recovered 10 out of the 16 activation interactions. We re-analyzed this dataset using CAT Bridge to test its performance with simple organisms. Utilizing the functions integrated within CAT Bridge and setting a time lag of 4, we calculated the association strengths between these molecules. The CCM method once again performed best, reproducing 14 out of 16 interactions.
Using CAT Bridge, we set a time lag of 3 to investigate the genes associated with Acetyl-CoA as the target metabolite. The CCM-based methods fared the best, and the results showed strong causality (greater than 0.9) with the aceE, aceF, and lpd genes, which are involved in the conversion of pyruvate to Acetyl-CoA (Figure 5A). These genes were also ranked in the top 100 in heuristic sorting and clustered together. Additionally, the acs and pta genes, which are involved in the conversion of acetate to

Powered by Editorial Manager® and ProduXion Manager® from Aries Systems Corporation

	Acetyl-CoA, also showed strong causality (0.840 and 0.721, respectively) but did not rank in the top 100 in heuristic sorting (Figure 5B). This suggests that in this study, the precursors for Acetyl-CoA may predominantly come from pyruvate."<br>We have also updated the Materials and Methods section (Lines 232-237): "The dataset for E. coli originated from a study on the interactions between metabolites and genes within the bacterium [33]. Researchers manipulated the culture conditions of E. coli, alternating between growth and starvation phases. They then collected the transcriptome and metabolome under these varying conditions to systematically explore how metabolites influence transcription factors. The dataset includes 29 time points where both transcriptomic and metabolomic information were concurrently obtained".<br><br>10.Does the tool generate a vector indicating many-to-many relationships or one-to-one relationships? In other words, does it reveal whether one gene is associated with many metabolites, and vice versa, or if it establishes a single gene-metabolite relationship? Please include this in the results section. Also, in the discussion section please include examples of application of these relationships in various fields e.g. metabolic engineering or cancer metabolism.<br>Author response: Thanks for your question. CAT Bridge generates a vector for each gene in relation to a given target metabolite. Therefore, for each run, many vectors will be generated, and each vector represents a single gene-to-metabolite relationship.<br>We have revised the Methods section to include this clarification: "The algorithm chosen by the user will then generate a vector for each gene representing one-to-one associations between the gene and the target metabolite (Lines 111-113)."<br>We also updated the Discussion section: "We believe that discovering associated gene-metabolite pairs will have practical applications in many fields. For example, in metabolic engineering, a single metabolite is often regulated by multiple genes, by comparing the association strength, one can infer which genes may play a dominant role under certain conditions, facilitating the development of targeted metabolic engineering strategies. Furthermore, in horticulture and breeding, identifying genes with causal relationships to key natural products, particularly in non-model organisms, can significantly aid in molecular breeding efforts, potentially leading to the development of new crop varieties with enhanced yields or desired characteristics (Lines 363-371)."<br><br>11.Table 1 compares the features of CAT Bridge with other available methods. It should encompass features provided by other tools that are not available in the author's tool, such as knowledge-driven integration or integration with a third-party database. Additionally, it should address the limitation posed by the requirement of time series data, which is not just a strength but also a challenge, particularly for epidemiology studies where multiple time series for gene expression may not be feasible.<br>Author response: Thanks for your suggestion. We have revised the Table 2 to provide a more comprehensive comparison of CAT Bridge with other available tools in the field to include a broader range of features. We highlighted not only the strengths of CAT Bridge but also notable features of other tools that are not present in CAT Bridge, include third-party database connectivity, knowledge-driven analysis, and integration. On the other hand, we would like to emphasize that CAT Bridge is currently designed to provide a solution for longitudinal multi-omics studies, rather than attempting to offer a universal solution for all multi-omics research. For fields where sampling across multiple time points is challenging, such as epidemiology, we recommend the use of established methods like Mendelian Randomization or structural equation modeling for causal inference or checking conformity with the Bradford Hill criteria.<br><br>12.Please use alternative phrases to "Self-generated data," such as "experimentally obtained data," to clarify that the author is utilizing data acquired in the lab to validate the tool. (e.g. line 42, 223, and 492).<br>Author response: Per your suggestion, we have revised the manuscript to replace the term "self-generated data" with "experimentally obtained data" to clarify that the data were acquired through laboratory experiments. This change has been applied to the specified lines and throughout the document where necessary (Lines 44, 251, 606, and Figure 1).<br>We would like to express our gratitude to both reviewers again for your meticulous review and insightful comments that help improve our manuscript.

Powered by Editorial Manager® and ProduXion Manager® from Aries Systems Corporation

Additional Information:	
Question	Response
Are you submitting this manuscript to a special series or article collection?	No
**Experimental design and statistics**<br><br>Full details of the experimental design and statistical methods used should be given in the Methods section, as detailed in our Minimum Standards Reporting Checklist. Information essential to interpreting the data presented should be made available in the figure legends.<br><br>Have you included all the information requested in your manuscript?	Yes
**Resources**<br><br>A description of all resources used, including antibodies, cell lines, animals and software tools, with enough information to allow them to be uniquely identified, should be included in the Methods section. Authors are strongly encouraged to cite Research Resource Identifiers (RRIDs) for antibodies, model organisms and tools, where possible.<br><br>Have you included the information requested as detailed in our Minimum Standards Reporting Checklist?	Yes
**Availability of data and materials**<br><br>All datasets and code on which the conclusions of the paper rely must be either included in your submission or deposited in publicly available repositories (where available and ethically appropriate), referencing such data using a unique identifier in the references and in the "Availability of Data and Materials" section of your manuscript.	Yes

Have you have met the above requirement as detailed in our Minimum Standards Reporting Checklist?	

Click here to access/download;Manuscript;comments_reply_catbridge_giga

北京大学 PEKING UNIVERSITY 现代农业研究院 INSTITUTE OF ADVANCED AGRICULTURAL SCIENCES

Dear Editor,

Enclosed is our revised manuscript entitled "CAT Bridge: An Efficient Toolkit for Gene-Metabolite Association Mining from Multi-Omics Data" that we would like to submit again as a Technical Note to **GigaScience** for consideration.

We are very grateful to you and the reviewers for giving us to valuable comments and suggestions to help improve our manuscript. We have been working on revising the manuscript carefully according to these comments and questions. Particularly we have addressed the reviewer concern about the LLM-based functionality and your requests on the RRID identifiers, which are described in our point-to-point response to all comments along with the revised manuscript. We trust that the revisions have substantially improved the manuscript, making it more suitable for publication in GigaScience.

Please let me know if further information is needed regarding the submission. We are looking forward to hearing from you.

Yours sincerely,

Li Guo, Ph.D.

Principal Investigator

Peking University Institute of Advanced Agricultural Sciences

Weifang, Shandong, China

li.guo@pku-iaas.edu.cn

Response to editor comments:

I'd like to emphasize that I completely share the major concerns of reviewer #1 regarding the use of an LLM for interpreting the data. The validity and factual correctness of this approach needs to be backed up with hard evidence, and limitations and caveats properly addressed. If a convincing validation of the LLM-based functionality is not possible, it might be preferable to omit or replace the GTP integration.

Author response: Thank you for your suggestion. We have thoroughly discussed this issue in our response to Reviewer #1. We have added a description of the method and on how to reduce AI hallucination problems by using appropriate prompt words and a low-temperature setting; we have also included a warning about the risks of using this feature. If the responses generated by the Large Language Model (LLM) are still considered misleading, we are prepared to completely remove this feature from CAT Bridge until the techniques are more mature.

In addition, please register any new software application in the bio.tools and SciCrunch.org databases to receive RRID (Research Resource Identification Initiative ID) and biotoolsID identifiers, and include these in your manuscript.

Author response: We have submitted CAT Bridge to receive the Research Resource Identification Initiative ID (SCR_025410) and biotoolsID (cat_bridge). And this information has been included in the Availability of source code and requirements section of our manuscript.

Response to reviewer comments:

Reviewer #1:

The authors introduce a useful tool (CAT Bridge) for integrating multiple causal and correlative analyses for multi-omics integration, which also includes a visualization and LLM component. The authors further provide two case studies (human and plant) illustrating the utility of CAT Bridge. I believe that this work should be published, as it contributes to the field of multi-omics analysis.

Author response: We are truly grateful for your positive acknowledgment of our work on the CAT Bridge tool. We believe CAT Bridge will serve as a beneficial resource for researchers in their analytical endeavors.

However, I am very concerned about the lack of description regarding the LLM. As explained by Mittelstadt et al (https://www.nature.com/articles/s41562-023-01744-0), LLMs do not always provide factual answers. The authors need to justify the use of the LLM to determine the relevance of a gene-metabolite association. In particular, the authors should add to the main text (or at least the supplementary) a detailed description of the prompt construction and should justify why this prompt is expected to result in factual information. Furthermore, the authors should discuss the caveats of using LLMs in this context, starting with the linked article above. I believe that the manuscript will only be publishable once this concern is addressed.

Author response: We are grateful for the reference provided, Mittelstadt *et al.* have thoroughly discussed the issue of LLMs regarding their potential to generate misleading information in the reference, and we acknowledge that current LLMs might not be absolutely trustworthy. However, we believe that the integration of an AI Agent is a valuable feature with acceptable limitations, especially for computational biologists who may lack domain knowledge of the molecular mechanisms underlying specific biological questions. Researchers can therefore prioritize the genes suggested by the LLM for downstream research and validation. As an additional layer of information to inspire further investigation, LLMs are not meant to replace empirical validation but to provide a starting point for experimental validation based on the synthesis of prior knowledge. Users of CAT Bridge can decide if they would like assistance from the AI agent, or trust their interpretation of the output from CAT Bridge.

To minimize the potential misguidance caused by hallucinations in the Master of Laws, we have set reasonable context settings and prompts, as well as a more conservative temperature setting (which will be elaborated on in the following content). We have also added additional disclaimers to the responses generated by the artificial intelligence, these will inform researchers that the results from the AI Agent are derived from an LLM's output, which does not ensure factual accuracy, and that manual verification is essential. Furthermore, this feature requires the user to obtain an OpenAI API key which is not provided in the web server version. We trust that users who engage with this feature generally possess a robust understanding of the operational mechanisms and inherent characteristics of LLMs' outputs.

In addition, we have added a new section detailing the LLM's methodology to the revised supplementary text: “The artificial intelligence (AI) Agent was developed utilizing the OpenAI API to integrate large language model (LLM) GPT-3.5 turbo. The prompts of the AI

agent are constructed based on the top 100 genes ranked from heuristic ranking, combining the genes and their functional annotations to create a list of formatted strings, followed by a question regarding their potential involvement in the synthesis of the target metabolite (Which one may be involved in the synthesis of [target metabolite]). To mitigate the risk of hallucinations or factual inaccuracies in the LLM's response, the 'system' role message contextualizes the AI as a knowledgeable biological chemist. Additionally, the temperature setting was lowered to 0.2, which resulted in more cautious and deterministic outputs. This approach minimizes the chances of deviating from factual data, favoring factual accuracy over creative but potentially speculative responses" (Lines 74-84).

We also incorporated a discourse on the caveats of utilizing LLMs into the Discussion: "LLM-based AI agents have shown a wide range of applications in various fields, but the hallucination of knowledge deficiency remains an issue that can mislead users. Although we have enhanced the credibility of the AI agent response via appropriate prompt and low temperature, this cannot be regarded as a substitute for professional knowledge and experimental verification, but merely serves as a starting point for verification, the definitive conclusions still require manual assessment by researchers" (Lines 387-392).

Finally, we also added this statement to the end of the AI agent response: "This response is generated by a Large Language Model (GPT 3.5 turbo) and should not be considered as a substitute for professional judgment. Users are advised to verify the accuracy of the information through rigorous literature review and, where necessary, experimental confirmation."

If the responses generated by the LLM are still regarded as misleading, we can completely remove this feature from CAT Bridge until the techniques mature.

In addition, the authors are recommended to address the following more minor concerns:

Implementation:

Your "example file" links at https://catbridge.work are broken. Please fix this.

Author response: We apologize for the inconvenience caused by the broken links, we have addressed the issue, and the links are now functional.

Abstract:

1. *Line 32: "Nevertheless, substantial challenges remain in determining the association between gene-metabolite pairs due to the complexity of cellular networks." This is not a*

clear statement. What about the complexity of cellular networks presents challenges in determining the associations?

Author response: We appreciate the opportunity to clarify the statement regarding the challenges presented by the complexity of cellular networks in determining gene-metabolite associations. The complexity arises due to several factors:

- Non-linear Relationships: Gene expression and metabolite production often exhibit non-linear dynamics, complex regulatory networks, and feedback loops, making it difficult to predict based on simple correlation-based models.

- Non-one-to-one Correspondence: Multiple genes can influence a single metabolite, and conversely, a single gene can affect multiple metabolites. This complexity makes it challenging to establish direct associations.

- Temporal Dynamics: The temporal dynamics in cellular networks pose challenges, as metabolite levels often lag behind changes in gene expression. This inherent delay complicates the direct determination of their association.

Therefore, we have revised the manuscript text accordingly: "Nevertheless, substantial challenges remain in determining the association between gene-metabolite pairs due to the non-linear and multifactorial interactions within cellular networks. The complexity arises from the interplay of multiple genes and metabolites, often involving feedback loops and time-dependent regulatory mechanisms that are not easily captured by traditional analysis methods" (Lines 32-36).

2. *Make sure you are using present tense consistently, not past tense (Line 39).*

Author response: Thank you for the suggestion. We have revised the manuscript text to ensure consistent use of the present tense.

3. *Please use the scientific name with the common name in parentheses as follows: Capsicum chinense (chili pepper). Use only the scientific name throughout the rest of the document (Line 41).*

Author response: Thanks for your suggestion. We have revised the manuscript to comply with the requested format for scientific names. The species is now initially presented as *Capsicum chinense* (chili pepper), and only the scientific name is used throughout the remainder of the manuscript.

Background:

1. Line 56: "Background" should not be plural.

Author response: Revised.

2. Lines 59-60: More comprehensive than what? Please elaborate here.

Author response: Thanks for the questions. We meant to say that it is more comprehensive than single-omic studies. The term "comprehensive" refers to the integration of multiple layers of molecular information and inter-level regulatory relationships. This integration enables us to have a broader picture of the biological system, thus determining whether observed changes at a molecular level from broader alterations across a metabolic pathway, or only regarding a specific molecule. Therefore, it provides a more systematic understanding of the biological mechanism, making it more comprehensive than single-omic studies.

We revised the manuscript as follows: "multi-omics data analysis is crucial for understanding intricate biological mechanisms from a more comprehensive perspective than single omics, this holistic analysis allows us to explore the interplay between different molecular levels." (Lines 59-62).

3. In Line 60, please include and familiarize yourself with the following reference: Eicher, T., G. Kinnebrew, A. Patt, K. Spencer, K. Ying, Q. Ma, R. Machiraju and E. A. Mathé (2020). "Metabolomics and Multi-Omics Integration: A Survey of Computational Methods and Resources." Metabolites 10: 202.

Author response: Thank you for recommending this review paper. We have carefully reviewed the literature and cited it, as it provides a comprehensive survey of computational methods and resources for metabolomics and multi-omics integration. It specifically discusses time series studies in omics, which is the main focus of our paper. This reference emphasizes the importance of addressing the lag issues between different omics layers. It uses Dynamic Time Warping (DTW), which is also integrated into CAT Bridge, as an example to illustrate how to align time points. Such specific data types are often overlooked in the research community's reviews (Lines 62).

4. Lines 67-68: Citation needed.

Author response: We have now included the necessary citations (doi: 10.1016/j.csbj.2021.09.001) in Lines 69-70 to support our statements, ensuring that our claims are well-substantiated.

5. Line 72: Please use the scientific name with the common name in parentheses.

Author response: Thank you for the reminder, we have updated our manuscript, now listing both the scientific and common names for two species: *Solanum lycopersicum* (tomato) and *Oryza sativa* (rice) (Line 74).

6. Lines 74-77: Citations needed.

Author response: We have now included the necessary citation (doi.org/10.1039/D2NP00032F and doi:10.3390/metabo10050202) in lines 77-80 to support this statement.

7. Lines 77-78: Give an example of biologically naïve conclusions from purely data-driven strategies.

Author response: Thanks for your suggestion! To help users understand the concept, we have included a classic example in our manuscript that shows how biologically naïve conclusions came from purely data-driven strategies. The manuscript has been revised as follows: "For example, a purely data-driven approach might incorrectly link low cholesterol levels with higher mortality rates, suggesting that lower cholesterol is detrimental. However, the actual cause is underlying diseases like cancer, which cause both low cholesterol and increased mortality" (Lines 83-86).

8. Line 78: Discuss how the machine learning models could address the drawbacks of the correlation models and vice-versa.

Author response: We apologize for the ambiguity. The "two methodologies" mentioned here refers to data-driven and knowledge-driven methods. To avoid ambiguity, we revised the sentence as "Therefore, integrating data-driven and knowledge-driven methodologies may offer a more comprehensive and accurate interpretation of multi-omics data. (Lines 87-88)"

The correlation models identify new connections and provide quantitative analysis, while knowledge-driven methods avoid many false positives caused by coincidental similarities to true functional gene expression patterns in situations where data is scarce. Thus, this combination improves generalizability and increases the interpretability of the model.

Materials and Methods:

1. It seems that CAT Bridge needs to be run on one metabolite at a time. In this case, I would not use the term "gene-metabolite pair association" in Line 104, but rather "associations between genes and the target metabolite".

Author response: Thank you for your suggestion, we have made the change in our manuscript (Lines 112-113). This revision enhances the clarity of our presentation and avoids any potential misunderstanding regarding the scope of CAT Bridge.

2. Line 115: Clearly state which of these methods are non-linear and which address the lag issue.

Author response: We have provided a detailed explanation of the methods in the revised manuscript (Table 1). Convergent Cross Mapping (CCM) and Dynamic Time Warping (DTW) are non-linear methods. CCM reconstructs the state space to detect causality in non-linear systems, while DTW allows flexible matching to handle non-linear alignments of time series data. Granger Causality (Granger) and Canonical Correlation Analysis (CCA) are inherently linear methods, although there are extensions such as non-linear Granger and kernel CCA that can address non-linear relationships. For handling time lag, CCM, Granger Causality, DTW, and the Cross-Correlation Function (CCF) are capable of addressing this issue. CCM and DTW inherently consider time lag through state space reconstruction and optimal matching, respectively. Granger Causality explicitly incorporates lagged values in its regression model, and CCF measures correlation at various time lags. Although standard CCA does not handle time lags, time-lagged variations of CCA can address this problem.

Table 1. Comparison of statistical methods by linearity and time lag consideration

Statistical Method	Linearity	Time Lag Consideration
Pearson Correlation Coefficient (Pearson)	Non-linear	No
Spearman Correlation Coefficient (Spearman)	Non-linear	No
Convergent Cross Mapping (CCM)	Non-linear	Yes
Granger Causality Test (Granger)	Linear	Yes
Canonical Correlation Analysis (CCA)	Linear	No
Dynamic-Time-Warping (DTW)	Non-linear	Yes
Cross-Correlation Function (CCF)	Linear	Yes

3. Line 136: Your figures are out of order (Figure 1B comes after Figure 2B).

Author response: Thanks for your comment. We apologize for the mistake. We have revised the manuscript to give the correct figure reference. The previous mention of "Figure 1B" has been revised to "Figure 2B".

4. Please take a look at the Minimum Standards Reporting Checklist (https://academic.oup.com/gigascience/pages/Minimum_Standards_of_Reporting_Checklist). In particular:

a. In the section starting at Line 153, list the number of seedlings used. b. Were all timepoints collected from all seedlings? List the total number of samples. c. How many mg were collected per sample (can use a range here)? d. 3 biological replicates per seedling? Give more detail here. e. What machine was used for the ultrasonic process? If frequency settings are permitted by the machine, list the settings used. f. How many of the 28 younger and 54 older adults had both transcriptome and metabolome data?

Author response: Thanks for your suggestion, we have revised our manuscript by considering this standard:

Line 167 to 188:

"To test the effectiveness of CAT Bridge across different species, especially its applicability to non-model organisms, we collected transcriptome sequencing and metabolic profiling data from *Capsicum chinense*, focusing on one of its trademark natural products, capsaicin. *C. chinense* seedlings were divided into three groups, each containing 15 seedlings, grown in a greenhouse of Peking University Institute of Advanced Agricultural Sciences with a controlled environment of 25°C temperature, a light-dark cycle of 16 hours light and 8 hours dark, and 70% relative humidity. The fruits of *C. chinense* were sampled at seven distinct time points, starting from the day of flowering, i.e. 0 day post-anthesis (DPA) during which flowers were collected, followed by fruit harvest on days 7, 16, 30, 50, 55, and 60 DPA. For each time point, we sampled 15 fruits from each group of seedlings, yielding a total of 45 sampling per time point. For each group, we utilized 1.0 mL of 70% aqueous methanol per sample, with a sample weight of approximately 50mg (aside from the 7 DPA samples which averaged 27.3mg). The samples were ground and freeze-dried in liquid nitrogen. Each sample was then extracted using 1.0 mL of 70% aqueous methanol for every 50 mg of sample. Following extraction, the samples underwent ultrasonic treatment using an Ultrasonic Cell Disruptor SCIENTZ-IID (Ningbo Scientz Biotechnology Co., LTD., China) at a frequency of

40 kHz for 10 minutes at room temperature. Standards were prepared as follows: a mixed standard solution, ranging from 20-50 μg/mL, was prepared using MS-grade methanol. For the amino acid standard solution, a 1 mg/mL stock solution was initially prepared in water, and then diluted with 50% methanol to achieve a final concentration of 50 μg/mL. Three biological sample replicates were utilized in the subsequent transcriptome and metabolome analyses."

Lines 226 - 229:

"The data was sourced from a published aging study that sampled transcriptome and metabolome data from 28 younger (20 to 25 years) and 54 older (55 to 66 years) female human donors. 13 time points are included in them where both the transcriptome and the metabolome data were detected."

5. Line 209: "Younger" and "older" are better terms.

Author response: We appreciate your suggestion. These terms have been updated throughout the manuscript to ensure consistency and precision in age group descriptions.

Results:

1. *Line 248: How does the AI agent analyze the functional annotations?*

Author response: Thanks for the question. The specific description of the AI agent is discussed in our response to your previous comment above. Specifically, for this case study, the top 100 genes in heuristic ranking ranked by the CCM-based method along with their functional annotations (generated by eggNOG-mapper) were fed to a GPT 3.5-based LLM. The context was set as a professional biochemist, with the inquiry focusing on which gene might be involved in the synthesis of capsaicin. The AI agent successfully located BC332_05016, a gene encoding Acyl-transferase, a key enzyme of capsaicin biosynthesis.

2. *Lines 281-282: "This illustrates the advantage of causal relationship modeling methods over traditional methods".*

Author response: Thanks for your correction, we have revised the manuscript accordingly (Line 308).

3. *Line 290: Please also include the updated IntLIM paper (IntLIM 2.0): Eicher, T., K. D. Spencer, J. K. Siddiqui, R. Machiraju and E. A. Mathe (2023). "IntLIM 2.0:*

identifying multi-omic relationships dependent on discrete or continuous phenotypic measurements." Bioinformatics Advances 3(1): vbad009.

Author response: Thank you for your suggestion on the citation. We have included this IntLIM2.0 paper to the revised manuscript (Line 339).

4. *Make sure the colors are consistent in Table 1.*

Author response: We have revised the table to ensure that the colors used are consistent.

5. *Line 156: The scientific name of the pepper species is inconsistent with other areas of the text.*

Author response: Thank you for your reminder, we have corrected the scientific name.

Figures:

1. *S1 should be provided as a table, not a figure.*

Author response: Thanks for your suggestion! This figure was used to show the visual output of CAT Bridge and contained content already shown in the supplementary table. Thus, we have removed Figure S1.

2. *Please make S2 larger. It is difficult to read.*

Author response: Revised as suggested.

3. *S3 needs labels (x axis, y axis, legend).*

Author response: Revised as suggested.

Thank you once again for your meticulous review of our work, especially for the detailed discussion regarding the LLMs and your attention to the scientific details within our manuscript, ensuring that it is presented with clarity, accuracy, and integrity.

Reviewer #2:

To the authors,

Thank you for the opportunity to review the manuscript GIGA-D-24-00083. The authors created a tool to predict association between genes and metabolites using various algorithms. The authors provide the tool as a web application, and as a python package. To get the reciprocal relationship between gene and metabolites, i.e. which metabolites can change which gene or vice versa, this tool can be a toolkit for the biologist or bioinformatician.

The tool has application specially the relationship between changes in genes and metabolites is not direct, many complex mechanisms exist e.g. epigenetic or polymorphism. So the tool can be alternate to other available tools.

Also, the manuscript brings the community focus on causal relationships instead of just correlation based relationships. The tool used temporal causality algorithms for predicting relationships between genes and metabolites.

Author response: Thank you for your positive comments, highlighting temporal causality between omics data and providing democratized analytical software were key objectives of our work.

However, I recommend major revisions before publication. Here are my reasons and comments for the revisions:

General issues with web accessibility and package installation :

1. *There are concerns about web accessibility, as indicated by web browsers flagging the connection as insecure. This may stem from geographical restrictions or the absence of HTTPS certification. Addressing these issues would ensure secure access to the server.*

Author response: Thanks for your excellent suggestion and for bringing up this issue. Currently, more and more browsers enforce stringent security protocols. Therefore, we have transitioned to using HTTPS to ensure a more secure protocol for accessing the server.

2. *Despite successful initiation of the client application from the git repository as a python module, no results were generated upon launching. It is suggested that the authors distribute the tool as a Docker image to facilitate seamless usage, eliminating concerns regarding dependencies and version compatibility.*

Author response: Thank you for the feedback and suggestion regarding the client application. We have created and distributed a Docker image, and it's currently accessible on Docker Hub (https://hub.docker.com/r/bowen172/cat-bridge). Additionally, we have provided complete environment information (https://github.com/Bowen999/CAT-Bridge/tree/main/client), enabling users to recreate the same environment using conda. This method is more straightforward and can avoid issues related to hardware architecture differences. By providing two files, it can avoid issues with version compatibility and environment setup.

Other comments:

1. *There are inconsistencies regarding data preprocessing. While the manuscript mentions that the tool will handle preprocessing, it also indicates that users need to provide processed files. Clarification is needed on whether preprocessing is required. It seems, the tool required preprocessed data.*

   Author response: Thanks for your comment. The software indeed requires preprocessed gene expression matrices and metabolite concentration matrices, instead of raw data. The preprocessing mentioned in the manuscript refers to handling missing values, standardization, and other such adjustments within these matrices. We have revised the manuscript to replace preprocessing" with "data processing" for clarity.

2. *For clarity use "causality and correlation" instead of "causality/correlation" algorithms.*

   Author response: We agree with you that this is a clearer terminology and avoids any ambiguity that might arise from the use of a slash. We have updated the manuscript to reflect this change. Thank you for the suggestion.

3. *Can the tool process any new temporal numerical data series, or does it specifically filter for genes? For instance, if I provide a list of proteins along with a list of genes, will I receive the association between them? It is suggested to include this in the discussion section.*

   Author response: We appreciate your insightful question regarding the tool's capacity to process various temporal numerical data series.

   Our paper primarily focuses on the discovery of associations between genes and metabolites. However, the algorithms integrated into CAT Bridge were originally applied in other fields. For example, Convergent Cross Mapping (CCM), the highest-performing algorithm in this study, was initially used in the ecological study. Similarly, the Granger causality test originated in an economics study. Theoretically, these algorithms have the potential to analyze time-series data of other molecular types, such as the associations between genes and proteins, proteins, and metabolites (and vice versa). To demonstrate the tool's versatility beyond gene-metabolite pairs, we have included a case study (Case Study 3) that investigates the relationship between metabolites and genes (more particularly, transcription factors). For further information, see our response to question 9 in this letter.

   We have also added the following content to the Discussion regarding this issue: “We tested the performance of CAT Bridge on the gene-to-metabolite and metabolite-to-gene tasks in

this research, since CAT Bridge is derived by integrating algorithms originating from different fields and its theoretical basis suggests it has generalizability, we believe that it can be used for investigating associations between other different molecular levels (such as gene to protein). Nonetheless, validating its performance across these different interactions is necessary for the conclusion due to the variations in complexity and regulatory timing at different molecular levels (Lines 380-384)."

4. *Does the tool offer the capability to generate a causal diagram or network from these vectors, thereby providing visual support for their assertion regarding the causal relationship between metabolites and genes? If the author is working in this direction, it is suggested that information can be added in the discussion section.*

Author response: Thanks for your question. Yes, we are actively exploring methods to visualize biological causal networks, including illustrating the time lag between metabolite concentrations and gene expression patterns, and integrating KEGG metabolic pathways information to construct molecular networks. We plan to include this functionality in our future work.

We have added the following statement to the Discussion: "Future work will involve determining response times between molecules at different levels, visualizing these lagged non-linear relationships, and constructing molecular networks based on temporal causality and metabolic pathways (Lines 400-402).

5. *What definition of causal relationship did the author use, and could they provide a citation for their definition. Predictability or any other criteria were used for causal relationships. Please include the definition or criteria in the introduction and method section.*

Author response: Thanks for your suggestion. The definition of causality utilized in this manuscript aligns with the concept of predictability, whereby causality is inferred based on the ability of one time series (gene expression) to predict another (metabolite concentration). This inference is implemented through the application of Convergent Cross Mapping and Granger Causality test.

To improve clarity, additional information was incorporated into the Background section with citations: "A more reliable solution is to use causality, which is inferred based on the ability of one time series (e.g., gene expression) to predict another (e.g., metabolite concentration), to replace the correlation coefficient (Lines 80-82)."

Similarly, the Methods section has been revised to include detailed descriptions: "including Granger Causality (Granger) and Convergent Cross Mapping (CCM) by evaluating the predictability of metabolite concentration from gene expression to represent causality (Lines 126-127)."

6. *What are the minimum or maximum time points (interval) for input files? e.g. will the tool work if I provide only two times points or If I provide 48 times points. Please include the information in the method section.*

Author response: Good question! In general, an increased number of sampling time points contributes to greater reliability of the results, as this mitigates the issue of false positive candidate genes that exhibit similar expression patterns to truly functional genes by coincidence. And in cases where time points are numerous, particularly with intensive sampling at short intervals, it is crucial to select a reasonable time lag. The example involving *E. coli*, as discussed in response to Question 9, effectively demonstrates this point.

7. *What is the influence of the number of time points on the vector relationship presented in the paper? Have any studies by the authors addressed this question? Please include the results and discussion.*

Author response: In our study, the influence of the number of time points primarily arises from the sampling interval, which affects the lag time settings, and whether the number of time points is sufficient to achieve comprehensive causal modeling results. The detail about this topic is discussed in Question 6 and Question 9.

8. *Could the authors clarify which heuristic algorithm was employed for ranking the genes? Additionally, can they elaborate on how their approach to gene ranking is heuristic rather than relying on mathematical optimization or algorithmic methods? Clarification on the term "heuristic" would be beneficial.*

Author response: The term "heuristic" refers to problem-solving techniques that employ practical methods to find solutions when classic methods are impractical. In our case, we have utilized a heuristic approach to rank genes based on their association with metabolites, rather than employing strict mathematical methods. The heuristic algorithm we employed for gene ranking integrates multiple factors, including the strength of the association between genes and metabolites as measured by various statistical methods, fold change values, and, optionally, gene function annotations. This composite scoring system is designed to prioritize genes that are more likely to be biologically relevant with the target gene. This ranking is not

the result of a single, universally optimal solution but rather a practical method to prioritize candidate genes for further investigation, this approach is flexible and can be adapted based on the availability of data and specific research questions.

We did not adopt strict mathematical methods but rather heuristic ranking for the following reasons:

- Complexity of Biological Systems: Biological systems are inherently complex, with many variables and interactions that are not fully captured by strict mathematical models, especially for models applicable to different species. Heuristic methods allow for flexibility in handling this complexity.

- Data Characteristics: The data from omics studies can be noisy and high-dimensional, often with missing values or incomplete information. Heuristic approaches can be more robust to these data characteristics compared to strict mathematical methods that may require complete and clean data.

- Scalability and Adaptability: Heuristic approaches are generally more adaptable to different research questions and can be scaled to various sizes of datasets without significant modifications.

9. Could the authors offer an example from studies conducted on yeast, E. coli, or other simple organisms, demonstrating how changes in gene sequences have readily been observed to affect metabolite levels? Please include that in the results section.

Author response: Thank you for the suggestion. Yes, in simple organisms, the regulation relationship between genes and metabolites also exhibits time lag and non-linear characteristics. Previous researchers have observed these phenomena in *E. coli* networks (https://doi.org/10.1089%2Fomi.2010.0074). To validate this and test CAT Bridge's performance on this task, we utilized an open-sourced transcriptomics and metabolomics dataset from *E. coli* (https://doi.org/10.1038/s41467-019-12474-1) to perform a new case study, which we have included as a new results section in the manuscript.

We have updated the Materials and Methods and Results section of our manuscript to include the following example:

Lines 232 to 237:

"The dataset for *E. coli* originated from a study on the interactions between metabolites and genes within the bacterium [33]. Researchers manipulated the culture conditions of *E. coli*, alternating between growth and starvation phases. They then collected the transcriptome and metabolome under these varying conditions to systematically explore how metabolites influence transcription factors. The dataset includes 29 time points where both transcriptomic and metabolomic information were concurrently obtained."

Lines 311 to 331:

"In the third study, we utilized a transcriptomic and metabolomic dataset from *E. coli* to demonstrate the capabilities of CAT Bridge in analyzing simple organisms. The dataset contains gene expression and metabolite concentration data across 29 time points, and it was originally generated by Lempp *et al.* to investigate the allosteric regulation of transcription factors (TFs) by metabolites. They constructed a "literature network" of known metabolite-TF interactions, including 16 interactions as activations of the TF by metabolites. Using Kinetic Correlation, which accommodates time lags, Lempp *et al.* recovered 10 out of the 16 activation interactions. We re-analyzed this dataset using CAT Bridge to test its performance with simple organisms. Utilizing the functions integrated within CAT Bridge and setting a time lag of 4, we calculated the association strengths between these molecules. The CCM method once again performed best, reproducing 14 out of 16 interactions.

Using CAT Bridge, we set a time lag of 3 to investigate the genes associated with Acetyl-CoA as the target metabolite. The CCM-based methods fared the best, and the results showed strong causality (greater than 0.9) with the aceE, aceF, and lpd genes, which are involved in the conversion of pyruvate to Acetyl-CoA (Figure 5A). These genes were also ranked in the top 100 in heuristic sorting and clustered together. Additionally, the acs and pta genes, which are involved in the conversion of acetate to Acetyl-CoA, also showed strong causality (0.840 and 0.721, respectively) but did not rank in the top 100 in heuristic sorting (Figure 5B). This suggests that in this study, the precursors for Acetyl-CoA may predominantly come from pyruvate."

We have also updated the Materials and Methods section (Lines 232-237): "The dataset for *E. coli* originated from a study on the interactions between metabolites and genes within the bacterium [33]. Researchers manipulated the culture conditions of *E. coli*, alternating between growth and starvation phases. They then collected the transcriptome and metabolome under these varying conditions to systematically explore how metabolites

influence transcription factors. The dataset includes 29 time points where both transcriptomic and metabolomic information were concurrently obtained”.

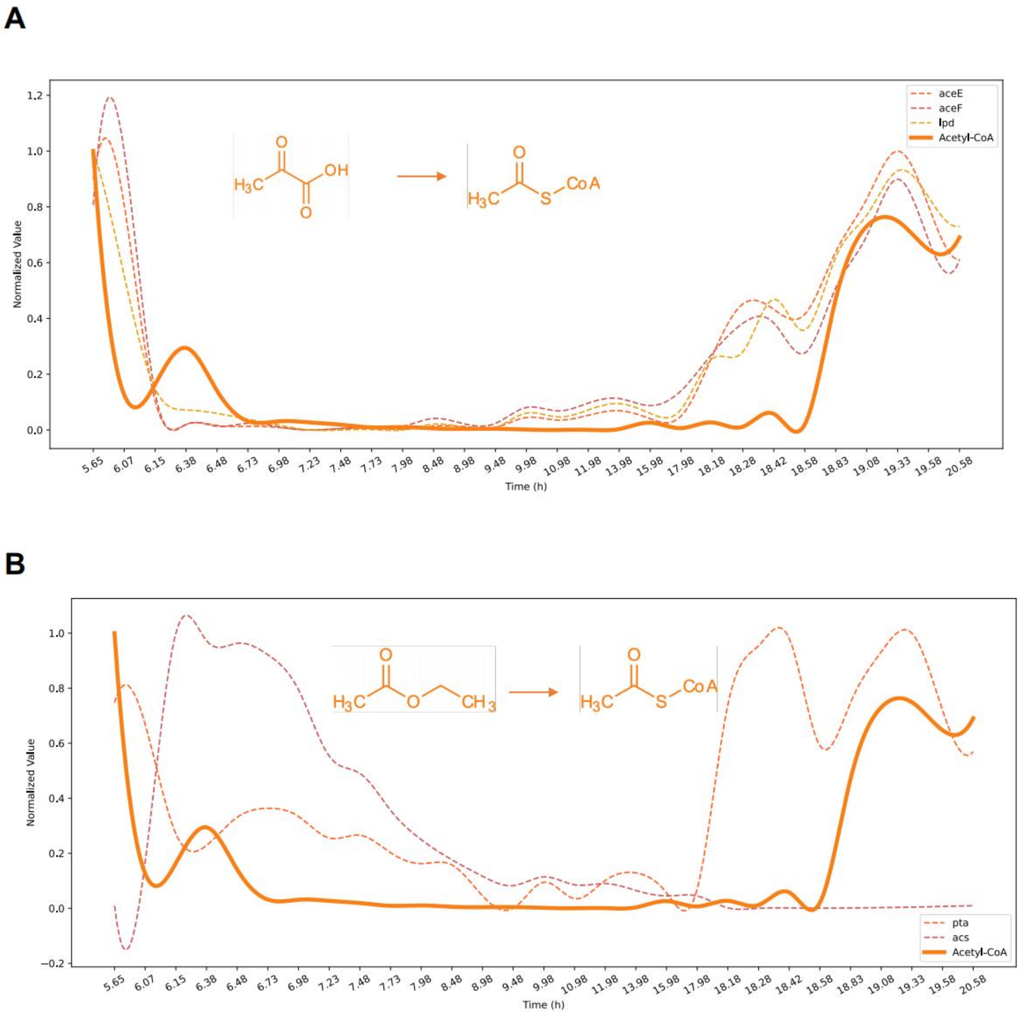

Figure 5. Expression Patterns of Acetyl-CoA Synthesis Genes and Acetyl-CoA Concentration. (A) Genes involved in the conversion of pyruvate to Acetyl-CoA. (B) Genes involved in the conversion of acetate to Acetyl-CoA.

We trust that this additional example strengthens our Results section and provides a clear demonstration of how CAT Bridge can effectively reveal gene-metabolite relationships in simple organisms.

10. *Does the tool generate a vector indicating many-to-many relationships or one-to-one relationships? In other words, does it reveal whether one gene is associated with many metabolites, and vice versa, or if it establishes a single gene-metabolite relationship? Please include this in the results section. Also, in the discussion section please include examples of application of these relationships in various fields e.g. metabolic engineering or cancer metabolism.*

Author response: Thanks for your question. CAT Bridge generates a vector for each gene in relation to a given target metabolite. Therefore, for each run, many vectors will be generated, and each vector represents a single gene-to-metabolite relationship.

We have revised the Methods section to include this clarification: "The algorithm chosen by the user will then generate a vector for each gene representing one-to-one associations between the gene and the target metabolite (Lines 111-113)."

We also updated the Discussion section: "We believe that discovering associated gene-metabolite pairs will have practical applications in many fields. For example, in metabolic engineering, a single metabolite is often regulated by multiple genes, by comparing the association strength, one can infer which genes may play a dominant role under certain conditions, facilitating the development of targeted metabolic engineering strategies. Furthermore, in horticulture and breeding, identifying genes with causal relationships to key natural products, particularly in non-model organisms, can significantly aid in molecular breeding efforts, potentially leading to the development of new crop varieties with enhanced yields or desired characteristics (Lines 363-371)."

11. *Table 1 compares the features of CAT Bridge with other available methods. It should encompass features provided by other tools that are not available in the author's tool, such as knowledge-driven integration or integration with a third-party database. Additionally, it should address the limitation posed by the requirement of time series data, which is not just a strength but also a challenge, particularly for epidemiology studies where multiple time series for gene expression may not be feasible.*

Author response: Thanks for your suggestion. We have revised the Table 2 to provide a more comprehensive comparison of CAT Bridge with other available tools in the field to include a broader range of features. We highlighted not only the strengths of CAT Bridge but also notable features of other tools that are not present in CAT Bridge, include third-party database connectivity, knowledge-driven analysis, and integration.

On the other hand, we would like to emphasize that CAT Bridge is currently designed to provide a solution for longitudinal multi-omics studies, rather than attempting to offer a universal solution for all multi-omics research. For fields where sampling across multiple time points is challenging, such as epidemiology, we recommend the use of established methods like Mendelian Randomization or structural equation modeling for causal inference or checking conformity with the Bradford Hill criteria.

Table 2. Comparison of CAT Bridge with other web-based multi-omics tools.

	CAT Bridge	Omics Analyst	3omics	IntLIM	CorDiffViz
Pre-processing	✓	✓	×	×	×
Visual analytics	✓	✓	✓	✓	✓
Cross-omics association	✓	×	✓	✓	✓
Cross-platform	✓	×	×	✓	✓
Longitudinal study	✓	×	×	×	×
Third-party database connectivity	×	×	×	×	×
Enrichment analysis	×	×	✓	×	×
AI agent	✓	×	×	×	×

12. *Please use alternative phrases to "Self-generated data," such as "experimentally obtained data," to clarify that the author is utilizing data acquired in the lab to validate the tool. (e.g. line 42, 223, and 492).*

Author response: Per your suggestion, we have revised the manuscript to replace the term "self-generated data" with "experimentally obtained data" to clarify that the data were acquired through laboratory experiments. This change has been applied to the specified lines and throughout the document where necessary (Lines 44, 251, 606, and Figure 1).

We would like to express our gratitude to both reviewers again for your meticulous review and insightful comments that help improve our manuscript.

Manuscript Click here to access/download;Manuscript;CAT Bridge.pdf

CAT Bridge: An Efficient Toolkit for Gene-Metabolite Association Mining from Multi-Omics Data

Authors

Bowen Yang[1,2], Tan Meng[1], Xinrui Wang[1], Jun Li[1], Shuang Zhao[3], Yingheng Wang[4], Shu Yi[1], Yi Zhou[1], Yi Zhang[1], Liang Li[2,3,*], Li Guo[1,*]

Affiliations

[1] Peking University Institute of Advanced Agricultural Sciences, Shandong Provincial Key Laboratory of Precision Molecular Crop Design and Breeding, Shandong Laboratory of Advanced Agricultural Sciences at Weifang, Weifang, 261325, China

[2] Department of Chemistry, University of Alberta, Edmonton, AB T6G 2G2, Canada

[3] The Metabolomics Innovation Centre, University of Alberta, Edmonton, AB T6G 1C9, Canada

[4] Department of Computer Science, Cornell University, Ithaca, NY 14853, USA

Author Email:

by8@ualberta.ca, mengtan_big_data@163.com, 1120230736@mail.nankai.edu.cn, jun.li@pku-iaas.edu.cn, szhao1@ualberta.ca, ys3702289@outlook.com, yw2349@cornell.edu, zhouyi_zoe@cau.edu.cn, 2022308310509@cau.edu.cn, liang.li@ualberta.ca, li.guo@pku-iaas.edu.cn

*Correspondence should be addressed to Guo Li. Tel: +86 13325308113; Email: li.guo@pku-iaas.edu.cn

*Correspondence may also be addressed to Liang Li. Tel: +1 (780) 492-3250; Email: liang.li@ualberta.ca

Running title: CAT Bridge

Abstract

Background: With advancements in sequencing and mass spectrometry technologies, multi-omics data can now be easily acquired for understanding complex biological systems. Nevertheless, substantial challenges remain in determining the association between gene-metabolite pairs due to the non-linear and multifactorial interactions within cellular networks. The complexity arises from the interplay of multiple genes and metabolites, often involving feedback loops and time-dependent regulatory mechanisms that are not easily captured by traditional analysis methods.

Findings: Here, we introduce Compounds And Transcripts Bridge (abbreviated as CAT Bridge, available at https://catbridge.work), a free user-friendly platform for longitudinal multi-omics analysis to efficiently identify transcripts associated with metabolites using time-series omics data. To evaluate the association of gene-metabolite pairs, CAT Bridge is a pioneering work benchmarking a set of statistical methods spanning causality estimation and correlation coefficient calculation for multi-omics analysis. Additionally, CAT Bridge features an artificial intelligence (AI) agent to assist users interpreting the association results.

Conclusions: We applied CAT Bridge to experimentally obtained *Capsicum chinense* (chili pepper) and public human and *Escherichia coli* (*E. coli*) time-series transcriptome and metabolome datasets. CAT Bridge successfully identified genes involved in the biosynthesis of capsaicin in *C. chinense*. Furthermore, case study results showed that the convergent cross mapping (CCM) method outperforms traditional approaches in longitudinal multi-omics analyses. CAT Bridge simplifies access to various established methods for longitudinal multi-omics analysis, and enables researchers to swiftly identify associated gene-metabolite pairs for further validation.

Keywords: Web server, gene-metabolite association, Multi-omics, Time series data, Causality

Background

Recent advancements in sequencing and mass spectrometry (MS) technologies have made the acquisition of multi-omics data more cost-efficient and feasible, multi-omics data analysis is crucial for understanding intricate biological mechanisms from a more comprehensive perspective than single omics, this holistic analysis allows us to explore the interplay between different molecular levels [1-4]. For integrated data analysis of transcriptomics and metabolomics, a crucial task is to examine the associated gene-metabolite pairs. Existing strategies bifurcate primarily into two classes: knowledge-driven approaches and data-driven approaches [5]. Knowledge-driven approaches have shown their inadequacies for non-model organisms due to the lack of knowledge, restrictions in revealing *de novo* mechanisms, and difficulties in quantifying and ranking their outcomes [5]. Data-driven strategies mainly depend on statistical methods that model the correlation of gene-metabolite pairs or sophisticated machine learning methods [6, 7]. However, due to the severe batch effects in omics data, machine learning approaches usually lack generalizability [8]. Meanwhile, they are also prone to overfit when applied to relatively small omics datasets, making them harder to transfer to different scenarios and less interpretable [5]. In terms of statistical methods, people usually calculate the correlation coefficient to match gene-compound pairs [9, 10] such as in the studies of the growth cycles of *Solanum lycopersicum* (tomato) [11] and *Oryza sativa* (rice) [12], where Pearson correlations were utilized to study metabolic regulatory networks by integrating transcriptomics and metabolomics data. However, these methods face reliability issues, especially when dealing with longitudinal omics data. This is because of the time lag in the expression of genes and metabolites, and the inherent complexity of biological systems, which is a dynamical system with non-linear interactions between different molecules [4, 13]. A more reliable solution is to use causality, which is inferred based on the ability of one time series (e.g., gene expression) to predict another (e.g., metabolite concentration), to replace the correlation coefficient [14, 15]. Furthermore, purely data-driven strategies can occasionally lead to biologically naive conclusions [9]. For example, a purely data-driven approach might incorrectly link low cholesterol levels with

higher mortality rates, suggesting that lower cholesterol is detrimental. However, the actual cause is underlying diseases like cancer, which cause both low cholesterol and increased mortality [16]. Therefore, integrating data-driven and knowledge-driven methodologies may offer a more comprehensive and accurate interpretation of multi-omics data.

To address the existing limitations, we have introduced Compounds And Transcripts Bridge (CAT Bridge), a comprehensive cross-platform toolkit that provides a novel analysis pipeline for integrative analysis linking upstream and downstream omics (typically transcriptomics and metabolomics). The novel pipeline encompasses three essential steps, data preprocessing, computing association between gene-metabolite pairs, and result presentation. For measuring the association, we integrated seven different statistical algorithms on causality estimation and correlation coefficient calculation and benchmarked them on human, plant, and microorganism datasets. It also offers three ways to display results that are generated from both data-driven approach and knowledge-driven approach, including common omics statistical analysis and visualization, heuristic ranking of candidate genes based on causality or correlation, and an AI agent driven by large language models (LLMs) to identify associated gene-metabolite pairs through prior knowledge **(Figure 1)**.

Besides, we offer three different access options for CAT Bridge, including a web server, a standalone application, and a Python library. We also provide a detailed tutorial and a sample dataset to help the users get started easily.

Materials and Methods

Overview of CAT Bridge

The workflow of CAT Bridge consists of three primary steps, (1) data processing; (2) statistical modeling (causality estimation and correlation coefficient calculation); (3) visualization and interpretation (**Figure 2A**). Users are required to upload two processed files, gene expression and metabolite concentration matrices, and specify a metabolite of interest as the target. After data processing, seven different causality and correlation algorithms are applied to measure the relationship between each gene and the target metabolite. The

algorithm chosen by the user will then generate a vector for each gene representing one-to-one associations between the gene and the target metabolite (**Figure 2B**). Subsequently, a vector magnitude is employed for heuristic ranking, with the top 100 genes being reviewed by the AI agent, to utilize prior knowledge to offer inspiration to users. Finally, commonly used omics visualization will be employed to assist users in interpreting the overall expression pattern and facilitating the selection of potential candidate genes.

Gene-metabolite association computing

For gene-metabolite pair identification such as inferring the biosynthetic genes for particular metabolites, correlation is often used to imply association, such as Spearman Correlation Coefficient (Spearman) and Pearson Correlation Coefficient (Pearson). However, such correlation-based methods have substantial limitations [9, 10] because they overlook the non-linearity and lag issues of gene expression leading to metabolite changes. Therefore, besides considering Spearman and Pearson, we have integrated into CAT Bridge various distinct statistical methods, including Granger Causality (Granger) and Convergent Cross Mapping (CCM) by evaluating the predictability of metabolite concentration from gene expression to represent causality, as well as Canonical Correlation Analysis (CCA), Dynamic-Time-Warping (DTW), Cross-Correlation Function (CCF) for calculating correlation coefficient (The implementation methods are provided in the Supplementary Text). These algorithms were based on different assumptions so that some of them allow compatibility with time series data and complex systems (**Table 1**). Among them, correlation-based strategies have been widely applied in genomics and multi-omics analysis [17-20]. The CCM and Granger, which estimate causality from time series data, are already used in some areas of biology such as ecology and neurobiology, but are overlooked in the omics analysis [14, 21-23]. Furthermore, our benchmarking results (as detailed in the Results section) suggest that in longitudinal multi-omics studies, causal relationships may provide a more accurate depiction of the association between genes and metabolites.

Table 1. Comparison of statistical methods by linearity and time lag consideration

Statistical Method	Linearity	Time Lag Consideration
Pearson Correlation Coefficient (Pearson)	Non-linear	No
Spearman Correlation Coefficient (Spearman)	Non-linear	No
Convergent Cross Mapping (CCM)	Non-linear	Yes
Granger Causality Test (Granger)	Linear	Yes
Canonical Correlation Analysis (CCA)	Linear	No
Dynamic-Time-Warping (DTW)	Non-linear	Yes
Cross-Correlation Function (CCF)	Linear	Yes

Heuristic ranking of candidate genes

Fold change (FC) is another measurement frequently used in omics analyses to identify differentially expressed genes [24]. CAT Bridge pinpoints the peak time of the target metabolite and calculates each gene's log2 normalized FC of this peak time point and the subsequent decline time point (that is, the next sampling time point after the peak time point) using DESeq2 [25]. Then, causality or correlation and FC are combined into a vector to represent the gene-metabolite pair. After the min-max normalization of values (The details are provided in the Supplementary Text), the magnitude of this vector is calculated as the CAT score (Figure 2B). This score heuristically ranks the strength of association between each gene and the metabolite. Users can filter putative genes based on thresholds (e.g., 0.5 for causality and correlation, 1 for normalized FC) or manually review them in descending order.

Knowledge-driven approaches and visualization

Optionally, if users provide a gene function annotations file, typically derived from homology annotations using tools like InterProScan [26] or eggNOG-mapper [27] for non-model organisms), the CAT score will be adjusted with an additional value. This value is determined by a scoring rule based on the gene's description. By default, genes annotated as enzymes

receive a score of 0.2, while those with unknown functions get a score of 0.1. Users can customize this scoring rule based on their specific requirements, depending on the presence of target annotations and their importance. Finally, the top 100 genes in heuristically ranking will be evaluated by the GPT-3.5 Turbo based AI agent, to identify putative genes on the gene's functional annotation and prior knowledge (The implementation methods are provided in the Supplementary Text). To enhance data interpretation, the CAT Bridge workflow offers a visual ranking of genes based on computation results, and also incorporates a spectrum of widely utilized graphical outputs, such as heatmap, and principal component analysis (PCA) plot, details can be found in the Supplementary Text.

Plant Material Cultivation and Sampling

To test the effectiveness of CAT Bridge across different species, especially its applicability to non-model organisms, we collected transcriptome sequencing and metabolic profiling data from *C. chinense*, focusing on one of its trademark natural products, capsaicin. *C. chinense* seedlings were divided into three groups, each containing 15 seedlings, grown in a greenhouse of Peking University Institute of Advanced Agricultural Sciences with a controlled environment of 25°C temperature, a light-dark cycle of 16 hours light and 8 hours dark, and 70% relative humidity. The fruits of *C. chinense* were sampled at seven distinct time points, starting from the day of flowering, i.e. 0 day post-anthesis (DPA) during which flowers were collected. Followed by fruit harvest on days 7, 16, 30, 50, 55, and 60 DPA. For each time point, we sampled 15 fruits from each group of seedlings, yielding a total of 45 sampling per time point. For each group, we utilized 1.0 mL of 70% aqueous methanol per sample, with a sample weight of approximately 50 mg (aside from the 7 DPA samples which averaged 27.3mg). The samples were ground and freeze-dried in liquid nitrogen. Each sample was then extracted using 1.0 mL of 70% aqueous methanol for every 50 mg of sample. Following extraction, the samples underwent ultrasonic treatment using an Ultrasonic Cell Disruptor SCIENTZ-IID (Ningbo Scientz Biotechnology Co., LTD., China) at a frequency of 40 kHz for 10 minutes at room temperature. Standards were prepared as follows: a mixed standard solution, ranging from 20-50 μg/mL, was prepared using MS-grade methanol. For

the amino acid standard solution, a 1 mg/mL stock solution was initially prepared in water, and then diluted with 50% methanol to achieve a final concentration of 50 μg/mL. Three biological sample replicates were utilized in the subsequent transcriptome and metabolome analyses.

Metabolome Profiling using HPLC-MS and Data Pre-processing

The metabolome profiling was carried out using untargeted metabolomics based on liquid chromatography coupled with mass spectrometry (LC-MS). The samples were filtered through a 0.22 μm membrane and transferred into the lining tube of a sampling vial. Subsequent centrifugation was carried out at 12000 rcf and 4°C for 10 minutes. The processed samples were then analyzed using Thermo Scientific Orbitrap Exploris™ 240 (Thermo Fisher Scientific, USA). Chromatographic separation was achieved on a T3 C18 (1.7 μm, 2.1 mm × 150 mm column, USA) maintained at 40°C. The mobile phase consisted of A: 1% formic acid in water and B: 1% formic acid in acetonitrile, with a flow rate of 300 μL/min. A 3 μL sample was injected at an autosampler temperature of 10°C. The elution gradient was set as follows: 0-2.5 min, 3-10% B; 2.5-6 min, 10-44% B; 6-14 min, 44-80% B; 14-20 min, 80-95% B; 20-23 min, 95% B; 23-23.1 min, 95-3% B; 23.1-28 min, 3% B. MS was performed using both positive and negative ion scans, with a precursor ion scan mode. The auxiliary gas heater temperature was set at 350°C, and the ion transfer tube temperature was also maintained at 350°C. The sheath gas flow rate and auxiliary gas flow rate were set to 35 arb and 15 arb, respectively. The voltages were set to 3.5 KV for the positive spectrum and 3.2 KV for the negative spectrum. For MS1, the scan resolution was 60000, with a scan range of 80-1200. For MS2, the scan resolution was 15000, with a stepped collision energy of 20, 40, and 60 eV. Metabolite identification and quantification were performed using the Compound Discoverer software 3.3 (Thermo Fisher Scientific, USA).

RNA extraction and transcriptome sequencing

Total RNA was isolated from the above collected plant materials using Trizol Reagent (Thermo Fisher, USA) following manufacturer recommended protocol. The quality of RNA

extracts was evaluated using RNA Nano 6000 Assay Kit of the Bioanalyzer 2100 system (Agilent Technologies, USA) following manufacturer's recommendation and samples with a RIN value >7 were used in downstream sequencing library construction and sequencing. The library construction was conducted using Illumina True-seq transcriptome kit (Illumina, USA) following standard protocols. Transcriptome sequencing was carried out by Novogene Co., Ltd. The sequencing reads were procured from the Illumina NovaSeq 6000 platform. For pre-processing, fastp [28] was employed to conduct quality control and clean the data. Subsequently, these reads were mapped to the *C. chinense* cultivar PI159236 genome [29] using STAR [30]. StringTie [31] was utilized to quantify and assess the expression levels of the genes that were successfully mapped. The biological function annotation of genes is obtained through the eggNOG-mapper.

Acquisition and processing of public datasets

We also collected human and *Escherichia coli* (*E. coli*) multi-omics data from public datasets to further examine the performance of CAT Bridge.

The human data was sourced from a published aging study [32] that sampled transcriptome and metabolome data from 28 younger (20 to 25 years) and 54 older (55 to 66 years) female human donors. 13 time points are included in them where both the transcriptome and the metabolome data were detected. We obtained the transcripts expression levels and the concentrations of glucose and fructose 6-phosphate for 13 donors, if multiple donor data were available at a single time point, the average value was used.

The dataset for *E. coli* originated from a study on the interactions between metabolites and genes within the bacterium [33]. Researchers manipulated the culture conditions of *E. coli*, alternating between growth and starvation phases. They then collected the transcriptome and metabolome under these varying conditions to systematically explore how metabolites influence transcription factors. The dataset includes 29 time points where both transcriptomic and metabolomic information were concurrently obtained.

Results

CAT Bridge provides a platform with a novel pipeline that allows for the rapid identification of putative genes for further investigation and validation. And three distinct usage modes are offered to cater to a wide range of user requirements. Firstly, it features a web server, designed for user-friendliness and accessibility, and is open to all users without any login requirements. This is particularly beneficial to those who are less familiar with programming languages. Secondly, a standalone application is available for users handling large data files, as it lifts the constraints on file sizes. Finally, a Python library is available for bioinformaticians with complete features and customizable workflows (The implementation of the software is provided in the Supplementary Text).

To showcase the utility and features of CAT Bridge, we applied it to both an experimentally obtained dataset collected from *C. chinense* and a publicly available human and *E. coli* dataset in two case studies. Our analysis revealed that in the context of longitudinal multi-omics, causality-based strategies tend to outperform those solely based on similarity. As such, we advocate for the adoption of causality rather than similarity in longitudinal multi-omics analysis such as co-mining the transcriptomics and metabolomics data. The capsaicin dataset generated in this study has been made open source for user exploration.

Case Study 1: Identifying genes associated with capsaicin biosynthesis in *C. chinense*

In our inaugural case study, we leveraged experimentally obtained non-model organism data to examine the performance of CAT Bridge. This data comprised the transcriptome and metabolome of *C. chinense* at seven different developmental stages after bloom (**Figure 3A**). Capsaicin, an important natural product produced by *C. chinense* that gives fruit pungency and has potential anti-cancer and analgesic activity [34], was selected as the target metabolite for this study. Time-series transcriptome and metabolic profiling of developing *C. chinense* fruits were used as input data to test CAT Bridge.

Through examination using the CCM method for hypothetical ranking, BC332_05016 encoding an Acyl-transferase was ranked first, regardless of whether an annotation file was

provided. The Result suggests that this gene was more likely to be the synthetic gene associated with capsaicin in *C. chinense* (**Figure 3B**). The complete heuristic ranking results are provided in Supplementary Table S1. BLAST search revealed that BC332_05016 was homologous of PUN1 (sequence identity: 100%), a.k.a AT3 (Acyl-transferase 3) or CS (capsaicin synthesis) gene [35]. Moreover, when common thresholds were applied for screening, only CCM passed the criteria. The causality modeled based on CCM was 0.55, implying a strong association between BC332_05016 and capsaicin. By contrast, the conventional Pearson correlation method produced a result of 0.08, which would fall below the commonly used threshold and potentially lead to an overlook of this gene-metabolite pair (**Figure 3C**). The AI agent also accurately found BC332_05016 among the top 100 genes based on functional annotation (**Figure 3D**). Furthermore, CAT Bridge visualization result showed that capsaicinoids such as nonivamide, dihydrocapsaicin, and homocapsaicin have high similarity to capsaicin (**Figure 3E**) and may play a significant role in response to the variable (**Figure 3F**). The rest of the visualization results are provided in the Supplementary Figures (**Figure S1-S4**). These results show that the CAT Bridge is a valuable tool in multi-omics analysis to reliably identify associated gene-metabolite pairs.

Case Study 2: Identifying association of gene-metabolite pairs in glycolysis

For case study 2, we utilized an open-source multi-omics dataset generated by previous aging research (Kuehne et al. 2017) [32]. After processing, 13 time points from the skin of younger and older people used for testing, we particularly compared different association modeling results from various statistical methods in CAT Bridge.

Using this dataset, Kuehne et al. found glycolysis altered activity in the upper body when aging, and hexokinase 2 (HK2) and phosphofructokinase (PFKP) two enzyme genes are reduced, whereas fructose bisphosphatase 1 (FBP1) and aldolase A (ALDOA) are increased in the older skin [32]. Because glycolysis is well-researched, and the study's focus was not identifying gene-metabolite pairs, they only compared the fold changes in genes and metabolites, without evaluating the strength of associations between gene-metabolite pairs.

To explore whether different statistical methods can identify known gene-metabolite pairs in glycolysis, we used this dataset to evaluate the relationship between key genes and glucose, fructose 6-phosphate, for fold change, we used the same comparison methods as Kuehne et al., that is older group divided by the younger group. The results show that CCM still performed the best, accurately identifying a strong negative correlation between the concentration of glucose and the expression of HK2; a strong positive correlation between fructose 1,6-bisphosphate and PFKP, and a weak negative correlation with ALDOA. However, only Spearman yielded the expected relationship between fructose 1,6-bisphosphate and FBP1 (**Figure 4A, B**). Additionally, previous studies did not identify hexokinase 1 (HK1) as a significant gene in the glucose change because its levels in the elderly were slightly higher than in younger individuals. However, CCM correctly identified this relationship. Taking the expression pattern of PFKP and fructose 1,6-bisphosphate as an example, we observed that changes in gene expression were always reflected in the metabolite concentration at the next time point, indicating a delay between metabolite and transcript responses. This illustrates the advantage of causal relationship modeling methods over traditional methods.

Case Study 3: metabolite-transcription factor interactions and Acetyl-CoA regulation

In the third study, we utilized a transcriptomic and metabolomic dataset from *E. coli* to demonstrate the capabilities of CAT Bridge in analyzing simple organisms. The dataset contains gene expression and metabolite concentration data across 29 time points, and it was originally generated by Lempp et al. to investigate the allosteric regulation of transcription factors (TFs) by metabolites [33]. They constructed a "literature network" of known metabolite-TF interactions, including 16 interactions as activations of the TF by metabolites. Using Kinetic Correlation, which accommodates time lags, Lempp et al. recovered 10 out of the 16 activation interactions. We re-analyzed this dataset using CAT Bridge to test its performance with simple organisms. Utilizing the functions integrated within CAT Bridge and setting a time lag of 4, we calculated the association strengths between these molecules. The CCM method once again performed best, reproducing 14 out of 16 interactions.

Focusing on Acetyl-CoA as the target metabolite and setting a time lag of 3, we investigated the associated genes. The CCM-based methods fared the best, and the results showed strong causality (greater than 0.9) with the aceE, aceF, and lpd genes, which are involved in the conversion of pyruvate to Acetyl-CoA (**Figure 5A**) [36]. These genes were also ranked in the top 100 in heuristic sorting and clustered together. Additionally, the acs and pta genes, which are involved in the conversion of acetate to Acetyl-CoA, [36], also showed strong causality (0.840 and 0.721, respectively) but did not rank in the top 100 in heuristic sorting (**Figure 5B**). The complete heuristic ranking results are provided in Supplementary Table S2. This suggests that in this study, the precursors for Acetyl-CoA may predominantly come from pyruvate.

These results demonstrate the CAT Bridge's potential to extract meaningful insights from multi-omics data across diverse species. By integrating time-series analysis methods, particularly CCM, it offers superior performance in longitudinal omics compared to common methods.

Comparison with other web-based tools

Table 2 displays the function coverage comparisons between CAT Bridge and other data-driven multi-omics analysis web-based tools, including OmicsAnalyst [5], 3omics [37], IntLIM [38, 39], and CorDiffViz [40]. In association identification, IntLIM, 3omics, and CorDiffViz integrate either Pearson or Spearman correlations, or both, to aid in the discovery of feature relationships. What sets CAT Bridge apart is its assembly of various algorithms that handle time-series data and causality, and incorporate an AI agent to inspire users. Notably, the performance of CCM has been found from three previous case studies to be potentially more suitable for longitudinal multi-omics analysis compared to traditional methods.

Table 2. Comparison of CAT Bridge with other web-based multi-omics tools.

	CAT Bridge	OmicsAnalyst	3omics	IntLIM	CorDiffViz
Pre-processing	√	√	×	×	×
Visual analytics	√	√	√	√	√
Cross-omics association	√	×	√	√	√
Cross-platform	√	×	×	√	√
Longitudinal study	√	×	×	×	×
Third-party database connectivity	×	×	×	×	×
Enrichment analysis	×	×	√	×	×
AI agent	√	×	×	×	×

Symbols indicating feature assessments: '√' denotes presence, '×' signifies absence. OmicsAnalyst: https://www.omicsanalyst.ca; 3omics: https://3omics.cmdm.tw; IntLIM: https://intlim.ncats.io; CorDiffViz: https://diffcornet.github.io/CorDiffViz/demo.html.

Discussion

In recent years, there has been a surge in multi-omics research. A critical aspect often overlooked in such studies is the unique nature of the longitudinal experimental design. Longitudinal omics analysis is particularly important in research on the developmental cycle of plants and investigations related to chronic diseases and aging [41-44]. However, many studies tend to use generic methodologies for analysis [11, 12]. This may inadvertently miss key discoveries. CAT Bridge provides a platform specifically for longitudinal multi-omics analysis, by drawing insights from disciplines where time series data is more prevalent and benchmarking them with data. Through the gene-metabolite causality and correlation modeling method, combined with visualization tools and AI assistance, researchers can more quickly identify putative genes for experimental validation. We believe that discovering associated gene-metabolite pairs will have practical applications in many fields. For example, in metabolic engineering, a single metabolite is often regulated by multiple genes, by comparing the association strength, one can infer which genes may play a dominant role

under certain conditions, facilitating the development of targeted metabolic engineering strategies. Furthermore, in horticulture and breeding, identifying genes with causal relationships to key natural products, particularly in non-model organisms, can significantly aid in molecular breeding efforts, potentially leading to the development of new crop varieties with enhanced yields or desired characteristics.

In three case studies, CCM showed its superiority compared to other methods, which is probably due to its modeling capability of dynamical systems. Thus, it can better capture the complex non-linear interactions within biological systems [21]. We advocate for modeling cause-and-effect relationships in longitudinal omics analyses, instead of more widely used Pearson or Spearman Correlations. However, this doesn't mean that CCM is always appropriate. Factors such as sampling intervals and the number of samples also need to be considered. More precise methods for estimating causality, as well as post-processing for vector represent gene-metabolite pairs are both required to explore and validate by using more data. We tested the performance of CAT Bridge on the gene-to-metabolite and metabolite-to-gene tasks in this research, since CAT Bridge is derived by integrating algorithms originating from different fields and its theoretical basis suggests it has generalizability, we believe that it can be used for investigating associations between other different molecular levels (such as gene to protein). Nonetheless, further validating its performance across these different interactions is necessary for the conclusion due to the variations in complexity and regulatory timing at different molecular levels. Additionally, LLM-based AI agents have shown a wide range of applications in various fields, but the hallucination of knowledge deficiency remains an issue that can mislead users [45, 46]. Although we have enhanced the credibility of the AI agent response via appropriate prompt and low temperature [46-48], this cannot be regarded as a substitute for professional knowledge and experimental verification, but merely serves as a starting point for verification, the definitive conclusions still require manual assessment by researchers.

Aside from computational methods, the reliability of analytical results is also influenced by experimental design and data acquisition methods. Increasing the number of sampling time points and setting a reasonable interval between them can enhance the credibility of the results. On the data acquisition front, it is recommended to annotate the transcriptome with an updated, high-quality reference genome, and employ advanced metabolomics techniques such as chemical isotope labeling liquid (CIL) LC-MS [49] to ensure a high coverage and more accurate relative quantification of metabolome.

Future work will involve determining response times between molecules at different levels, visualizing these lagged non-linear relationships, and constructing molecular networks based on temporal causality and metabolic pathways.

Availability of source code and requirements

Project name: CAT Bridge

Project home page: https://catbridge.work

GitHub page: https://github.com/Bowen999/CAT-Bridge

Operating system(s): Platform independent

Programming language: Python, R, HTM, CSS, JavaScript

License: CC0 1.0 Public Domain Dedication

Biotools: cat_bridge

RRID: SCR_025410

Additional Files

Supplementary Text 1. Extended experimental procedure

Supplementary Table S1. Heuristic ranking results of Case Study 1

Supplementary Table S2 Heuristic ranking results of Case Study 3

Supplementary Fig. S1. PCA plot from the test results of Case Study 1

(A) PCA plot of transcriptomics. (B) PCA plot of Metabolomics. (C) PCA plot of integrated multi-omics

Supplementary Fig. S2. Heatmap from the test results of Case Study 1

(A) Heatmap of transcriptomics. (B) Heatmap of Metabolomics.

Supplementary Fig. S3. Volcano plot (peak vs decline points) from the test result of Case Study 1

Supplementary Fig. S4. VIP plot of gene from the test result of Case Study 1

Author Contribution

B Yang: Conceptualization, Software, Methodology, Visualization, Investigation, Writing—original draft. T Meng: Software. X Wang: Visualization. J Li: Investigation. S Zhao: Writing—review & editing. Y Wang: Methodology, Writing—review & editing. S Yi: Investigation. Y Zhou: Software. Y Zhang: Software. L Li: Supervision. L Guo: Conceptualization, Writing—review & editing, Supervision, Funding acquisition. All authors have read and agreed to the published version of the manuscript.

Funding

This work was supported by the Key R&D Program of Shandong Province (Grant No. ZR202211070163) and Natural Science Foundation for Distinguished Young Scholars (Grant No. ZR2023JQ010) of Shandong Province. LG is also supported by Taishan Scholars Program of Shandong Province.

Acknowledgement

We would like to thank the Bioinformatics Platform at Peking University Institute of Advanced Agricultural Sciences for providing the high-performance computing resources.

Data availability

The CAT Bridge web server is available at https://www.catbridge.work, it is open and free for all users and there is no login requirement. The source code used for CAT Bridge is available on FigShare at https://doi.org/10.6084/m9.figshare.25044854.v3.

The sequencing data for case study 1 is available in the Small Read Archive (SRA) at http://www.ncbi.nlm.nih.gov/sra, under the BioProject accession code PRJNA1030882, and the metabolome data for this case study has been deposited at the Metabolomics Workbench

(https://www.metabolomicsworkbench.org) with the study ID ST003172. The sequencing data of case study 2 was obtained from Gene Expression Omnibus (GEO), with the accession number GSE85358. The sequencing data of case study 3 was obtained from GEO with the accession code GSE131992, and metabolomics data was sourced from the MetaboLights database with the accession code MTBLS1044.

Competing interests

The authors have declared no competing interests.

Uncategorized References

1. Wörheide MA, Krumsiek J, Kastenmüller G and Arnold M. Multi-omics integration in biomedical research - A metabolomics-centric review. Anal Chim Acta. 2021;1141:144-62. doi:10.1016/j.aca.2020.10.038.
2. Hasin Y, Seldin M and Lusis A. Multi-omics approaches to disease. Genome Biology. 2017;18 1:83. doi:10.1186/s13059-017-1215-1.
3. Subramanian I, Verma S, Kumar S, Jere A and Anamika K. Multi-omics Data Integration, Interpretation, and Its Application. Bioinform Biol Insights. 2020;14:1177932219899051. doi:10.1177/1177932219899051.
4. Eicher T, Kinnebrew G, Patt A, Spencer K, Ying K, Ma Q, et al. Metabolomics and Multi-Omics Integration: A Survey of Computational Methods and Resources. Metabolites. 2020;10 5:202.
5. Zhou G, Ewald J and Xia J. OmicsAnalyst: a comprehensive web-based platform for visual analytics of multi-omics data. Nucleic Acids Research. 2021;49 W1:W476-W82. doi:10.1093/nar/gkab394.
6. Krassowski M, Das V, Sahu SK and Misra BB. State of the Field in Multi-Omics Research: From Computational Needs to Data Mining and Sharing. Front Genet. 2020;11:610798. doi:10.3389/fgene.2020.610798.
7. Athieniti E and Spyrou GM. A guide to multi-omics data collection and integration for translational medicine. Computational and Structural Biotechnology Journal. 2023;21:134-49. doi:https://doi.org/10.1016/j.csbj.2022.11.050.
8. Albaradei S, Thafar M, Alsaedi A, Van Neste C, Gojobori T, Essack M, et al. Machine learning and deep learning methods that use omics data for metastasis prediction. Comput Struct Biotechnol J. 2021;19:5008-18. doi:10.1016/j.csbj.2021.09.001.
9. Cavill R, Jennen D, Kleinjans J and Briedé JJ. Transcriptomic and metabolomic data integration. Briefings in Bioinformatics. 2015;17 5:891-901. doi:10.1093/bib/bbv090.
10. Chong J and Xia J. Computational Approaches for Integrative Analysis of the Metabolome and Microbiome. Metabolites. 2017;7 4:62.
11. Li Y, Chen Y, Zhou L, You S, Deng H, Chen Y, et al. MicroTom metabolic network: rewiring tomato metabolic regulatory network throughout the growth cycle. Molecular plant. 2020;13 8:1203-18.

12. Yang C, Shen S, Zhou S, Li Y, Mao Y, Zhou J, et al. Rice metabolic regulatory network spanning the entire life cycle. Molecular Plant. 2022;15 2:258-75.
13. Singh KS, van der Hooft JJJ, van Wees SCM and Medema MH. Integrative omics approaches for biosynthetic pathway discovery in plants. Natural Product Reports. 2022;39 9:1876-96. doi:10.1039/D2NP00032F.
14. Ye H, Deyle ER, Gilarranz LJ and Sugihara G. Distinguishing time-delayed causal interactions using convergent cross mapping. Scientific Reports. 2015;5 1:14750. doi:10.1038/srep14750.
15. Yuan AE and Shou W. Data-driven causal analysis of observational biological time series. Elife. 2022;11 doi:10.7554/eLife.72518.
16. Sattar N and Preiss D. Reverse Causality in Cardiovascular Epidemiological Research. Circulation. 2017;135 24:2369-72. doi:doi:10.1161/CIRCULATIONAHA.117.028307.
17. Rockwood AL, Crockett DK, Oliphant JR and Elenitoba-Johnson KS. Sequence alignment by cross-correlation. J Biomol Tech. 2005;16 4:453-8.
18. Skutkova H, Vitek M, Babula P, Kizek R and Provaznik I. Classification of genomic signals using dynamic time warping. BMC Bioinformatics. 2013;14 10:S1. doi:10.1186/1471-2105-14-S10-S1.
19. Seoane JA, Campbell C, Day IN, Casas JP and Gaunt TR. Canonical correlation analysis for gene-based pleiotropy discovery. PLoS Comput Biol. 2014;10 10:e1003876. doi:10.1371/journal.pcbi.1003876.
20. Jiang MZ, Aguet F, Ardlie K, Chen J, Cornell E, Cruz D, et al. Canonical correlation analysis for multi-omics: Application to cross-cohort analysis. PLoS Genet. 2023;19 5:e1010517. doi:10.1371/journal.pgen.1010517.
21. Yuan AE and Shou W. Data-driven causal analysis of observational biological time series. eLife. 2022;11:e72518. doi:10.7554/eLife.72518.
22. Heerah S, Molinari R, Guerrier S and Marshall-Colon A. Granger-causal testing for irregularly sampled time series with application to nitrogen signalling in Arabidopsis. Bioinformatics. 2021;37 16:2450-60. doi:10.1093/bioinformatics/btab126.
23. Stokes PA and Purdon PL. A study of problems encountered in Granger causality analysis from a neuroscience perspective. Proc Natl Acad Sci U S A. 2017;114 34:E7063-e72. doi:10.1073/pnas.1704663114.
24. Arora S, Pattwell SS, Holland EC and Bolouri H. Variability in estimated gene expression among commonly used RNA-seq pipelines. Scientific Reports. 2020;10 1:2734. doi:10.1038/s41598-020-59516-z.
25. Love MI, Huber W and Anders S. Moderated estimation of fold change and dispersion for RNA-seq data with DESeq2. Genome Biology. 2014;15 12:550. doi:10.1186/s13059-014-0550-8.
26. Ye J, Coulouris G, Zaretskaya I, Cutcutache I, Rozen S and Madden TL. Primer-BLAST: a tool to design target-specific primers for polymerase chain reaction. BMC Bioinformatics. 2012;13:134. doi:10.1186/1471-2105-13-134.
27. Cantalapiedra CP, Hernández-Plaza A, Letunic I, Bork P and Huerta-Cepas J.

eggNOG-mapper v2: Functional Annotation, Orthology Assignments, and Domain Prediction at the Metagenomic Scale. Molecular Biology and Evolution. 2021;38 12:5825-9. doi:10.1093/molbev/msab293.

28. Chen S, Zhou Y, Chen Y and Gu J. fastp: an ultra-fast all-in-one FASTQ preprocessor. Bioinformatics. 2018;34 17:i884-i90. doi:10.1093/bioinformatics/bty560.

29. Kim S, Park J, Yeom SI, Kim YM, Seo E, Kim KT, et al. New reference genome sequences of hot pepper reveal the massive evolution of plant disease-resistance genes by retroduplication. Genome Biol. 2017;18 1:210. doi:10.1186/s13059-017-1341-9.

30. Dobin A, Davis CA, Schlesinger F, Drenkow J, Zaleski C, Jha S, et al. STAR: ultrafast universal RNA-seq aligner. Bioinformatics. 2013;29 1:15-21. doi:10.1093/bioinformatics/bts635.

31. Pertea M, Pertea GM, Antonescu CM, Chang T-C, Mendell JT and Salzberg SL. StringTie enables improved reconstruction of a transcriptome from RNA-seq reads. Nature Biotechnology. 2015;33 3:290-5. doi:10.1038/nbt.3122.

32. Kuehne A, Hildebrand J, Soehle J, Wenck H, Terstegen L, Gallinat S, et al. An integrative metabolomics and transcriptomics study to identify metabolic alterations in aged skin of humans in vivo. BMC Genomics. 2017;18 1:169. doi:10.1186/s12864-017-3547-3.

33. Lempp M, Farke N, Kuntz M, Freibert SA, Lill R and Link H. Systematic identification of metabolites controlling gene expression in E. coli. Nature Communications. 2019;10 1:4463. doi:10.1038/s41467-019-12474-1.

34. Fattori V, Hohmann MS, Rossaneis AC, Pinho-Ribeiro FA and Verri WA. Capsaicin: Current Understanding of Its Mechanisms and Therapy of Pain and Other Pre-Clinical and Clinical Uses. Molecules. 2016;21 7 doi:10.3390/molecules21070844.

35. Kim S, Park M, Yeom S-I, Kim Y-M, Lee JM, Lee H-A, et al. Genome sequence of the hot pepper provides insights into the evolution of pungency in Capsicum species. Nature Genetics. 2014;46 3:270-8. doi:10.1038/ng.2877.

36. Chiang C-J, Ho Y-J, Hu M-C and Chao Y-P. Rewiring of glycerol metabolism in Escherichia coli for effective production of recombinant proteins. Biotechnology for Biofuels. 2020;13 1:205. doi:10.1186/s13068-020-01848-z.

37. Kuo TC, Tian TF and Tseng YJ. 3Omics: a web-based systems biology tool for analysis, integration and visualization of human transcriptomic, proteomic and metabolomic data. BMC Syst Biol. 2013;7:64. doi:10.1186/1752-0509-7-64.

38. Siddiqui JK, Baskin E, Liu M, Cantemir-Stone CZ, Zhang B, Bonneville R, et al. IntLIM: integration using linear models of metabolomics and gene expression data. BMC Bioinformatics. 2018;19 1:81. doi:10.1186/s12859-018-2085-6.

39. Eicher T, Spencer KD, Siddiqui JK, Machiraju R and Mathé EA. IntLIM 2.0: identifying multi-omic relationships dependent on discrete or continuous phenotypic measurements. Bioinformatics Advances. 2023;3 1 doi:10.1093/bioadv/vbad009.

40. Yu S, Drton M, Promislow DEL and Shojaie A. CorDiffViz: an R package for visualizing multi-omics differential correlation networks. BMC Bioinformatics. 2021;22 1:486. doi:10.1186/s12859-021-04383-2.

41. Kudryashova KS, Burka K, Kulaga AY, Vorobyeva NS and Kennedy BK. Aging Biomarkers: From Functional Tests to Multi-Omics Approaches. Proteomics. 2020;20 5-6:e1900408. doi:10.1002/pmic.201900408.
42. Cellerino A and Ori A. What have we learned on aging from omics studies? Seminars in Cell & Developmental Biology. 2017;70:177-89. doi:https://doi.org/10.1016/j.semcdb.2017.06.012.
43. Allegri M, Gregori MD, Minella CE, Klersy C, Wang W, Sim M, et al. 'Omics' biomarkers associated with chronic low back pain: protocol of a retrospective longitudinal study. BMJ Open. 2016;6 10:e012070. doi:10.1136/bmjopen-2016-012070.
44. Mars RAT, Yang Y, Ward T, Houtti M, Priya S, Lekatz HR, et al. Longitudinal Multi-omics Reveals Subset-Specific Mechanisms Underlying Irritable Bowel Syndrome. Cell. 2020;182 6:1460-73.e17. doi:https://doi.org/10.1016/j.cell.2020.08.007.
45. Mittelstadt B, Wachter S and Russell C. To protect science, we must use LLMs as zero-shot translators. Nature Human Behaviour. 2023;7 11:1830-2. doi:10.1038/s41562-023-01744-0.
46. Rosoł M, Gąsior JS, Łaba J, Korzeniewski K and Młyńczak M. Evaluation of the performance of GPT-3.5 and GPT-4 on the Polish Medical Final Examination. Scientific Reports. 2023;13 1:20512. doi:10.1038/s41598-023-46995-z.
47. Antaki F, Milad D, Chia MA, Giguère C-É, Touma S, El-Khoury J, et al. Capabilities of GPT-4 in ophthalmology: an analysis of model entropy and progress towards human-level medical question answering. British Journal of Ophthalmology. 2023:bjo-2023-324438. doi:10.1136/bjo-2023-324438.
48. Miotto M, Rossberg N and Kleinberg B. Who is GPT-3? An exploration of personality, values and demographics. arXiv preprint arXiv:220914338. 2022.
49. Zhao S, Li H, Han W, Chan W and Li L. Metabolomic Coverage of Chemical-Group-Submetabolome Analysis: Group Classification and Four-Channel Chemical Isotope Labeling LC-MS. Anal Chem. 2019;91 18:12108-15. doi:10.1021/acs.analchem.9b03431.

Figure Legends

Figure 1. The architecture of CAT Bridge project

Benchmark data come from publicly available datasets and our experimentally obtained *C. chinense* dataset, including gene expression and metabolite concentration matrices. The construction of CAT Bridge relies on Python, R, HTML/CSS, and JavaScript, and provides three modes of usage: web server, standalone program, and Python library. To assist in the discovery of associated gene-metabolite pairs, it provides results from three perspectives: statistical analysis, AI agent-generated responses, and data visualization.

Figure 2. The features and overall workflow of CAT Bridge

(A) The workflow of CAT Bridge consists of three primary steps: data preprocessing, estimation of cause-effect relationships or computation of correlation coefficient, and the presentation of results, which includes visualization, heuristic ranking, and responses from an AI agent. (B) The computation of CAT Bridge involves: extracting the target metabolite from the metabolite concentration matrix, and pinpointing the time point of its maximum concentration as the peak time. Next, the causality or correlation between each gene in the gene expression matrix and the target metabolite is obtained. Then along with the fold change between the peak and decline time points to compose a vector to represent the association between gene-metabolite pairs.

Figure 3. **Application of CAT Bridge to mine transcript-capsaicin association**

(A) Diagram showing the time points sampled for transcriptome and metabolic profiling during fruit development of *C. chinense* in case study 1. (B) Heuristic ranking produced using the CCM-based method. (C) Comparative values across different methods. For unnormalized values: red indicates a strong association; original denotes medium association; blue suggests values that are below the commonly used threshold, show no association, or are negatively associated (depending on the method); light blue means this method does not adhere to a common threshold. For normalized values: red signifies values that are high after min-max normalization; blue represents low normalized values. (D) Interpretation results derived from the AI agent (E) The correlation network of capsaicin. (F) The significance of metabolites.

Figure 4. Application of CAT Bridge to mine transcript-metabolite associations in glycolysis pathway

(A) Left side: the expression patterns of functional genes and their corresponding target metabolites. Right side: the strength of associations was evaluated using different methods, red denotes evaluation outcomes that align with known pathway information under typical thresholds (e.g., a correlation coefficient < -0.5 indicating a strong negative association), whereas grey signifies inconsistent or unsuitable. (B) Part of the glycolysis pathway.

Figure 5. Expression Patterns of Acetyl-CoA Synthesis Genes and Acetyl-CoA Concentration

(A) Genes involved in the conversion of pyruvate to Acetyl-CoA. (B) Genes involved in the conversion of acetate to Acetyl-CoA.

Click here to access/download;Figure;Figure 1.pdf

Figure 1

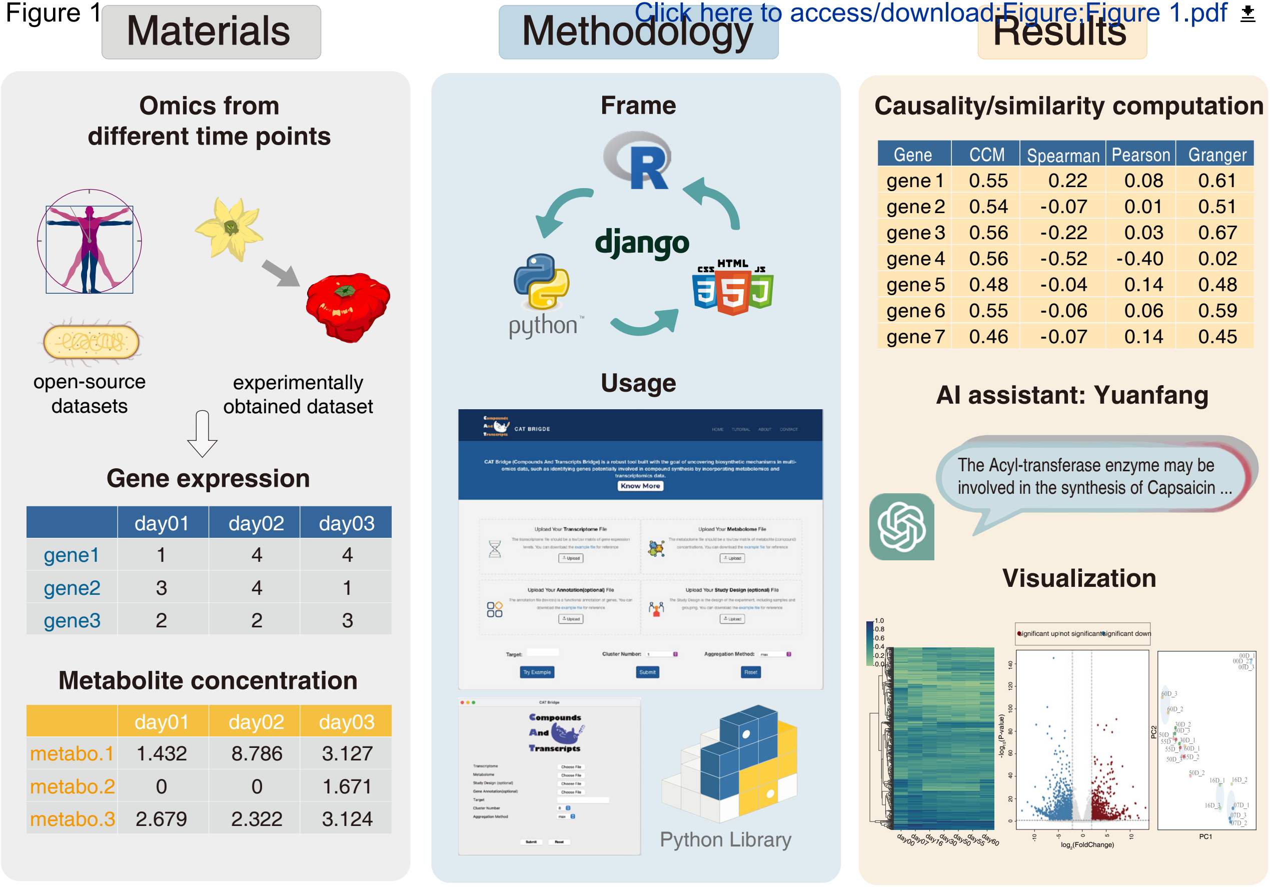

Figure 2

Click here to access/download;Figure;Figure 2.pdf

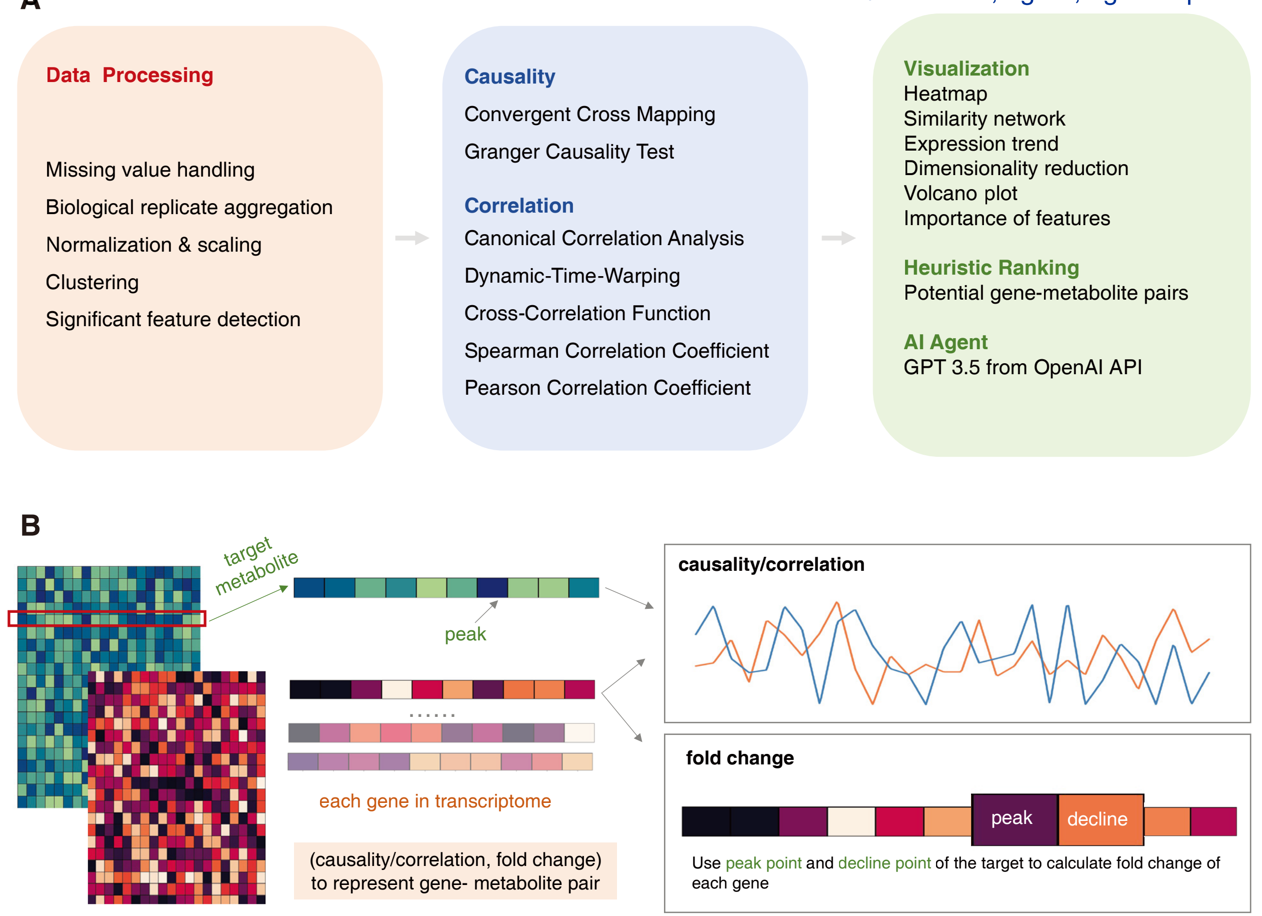

Figure 3

Click here to access/download;Figure;Figure 3.pdf

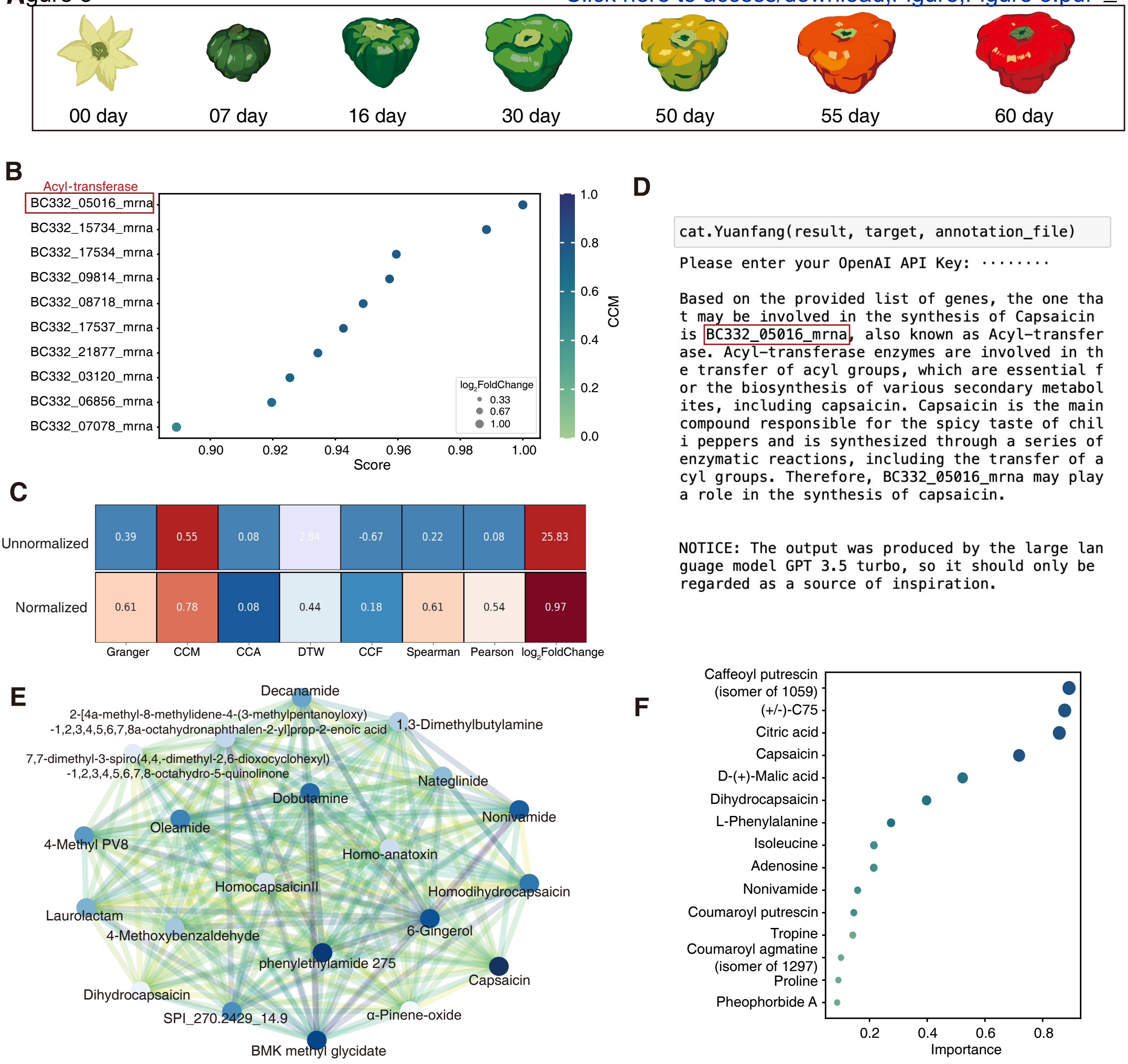

Figure 4

Click here to access/download;Figure;Figure 4.pdf

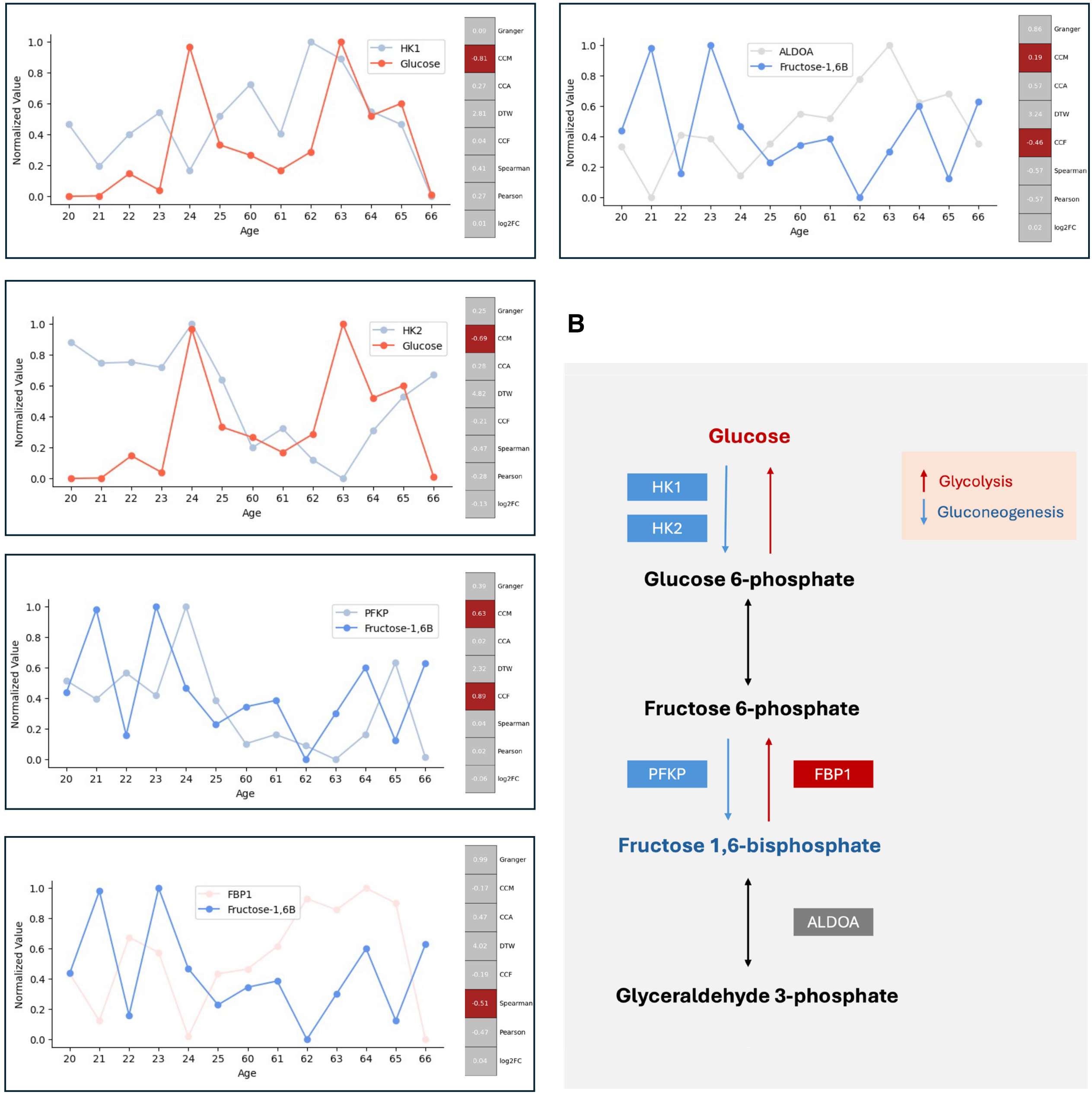

Figure 5

Click here to access/download;Figure;Figure 5.pdf

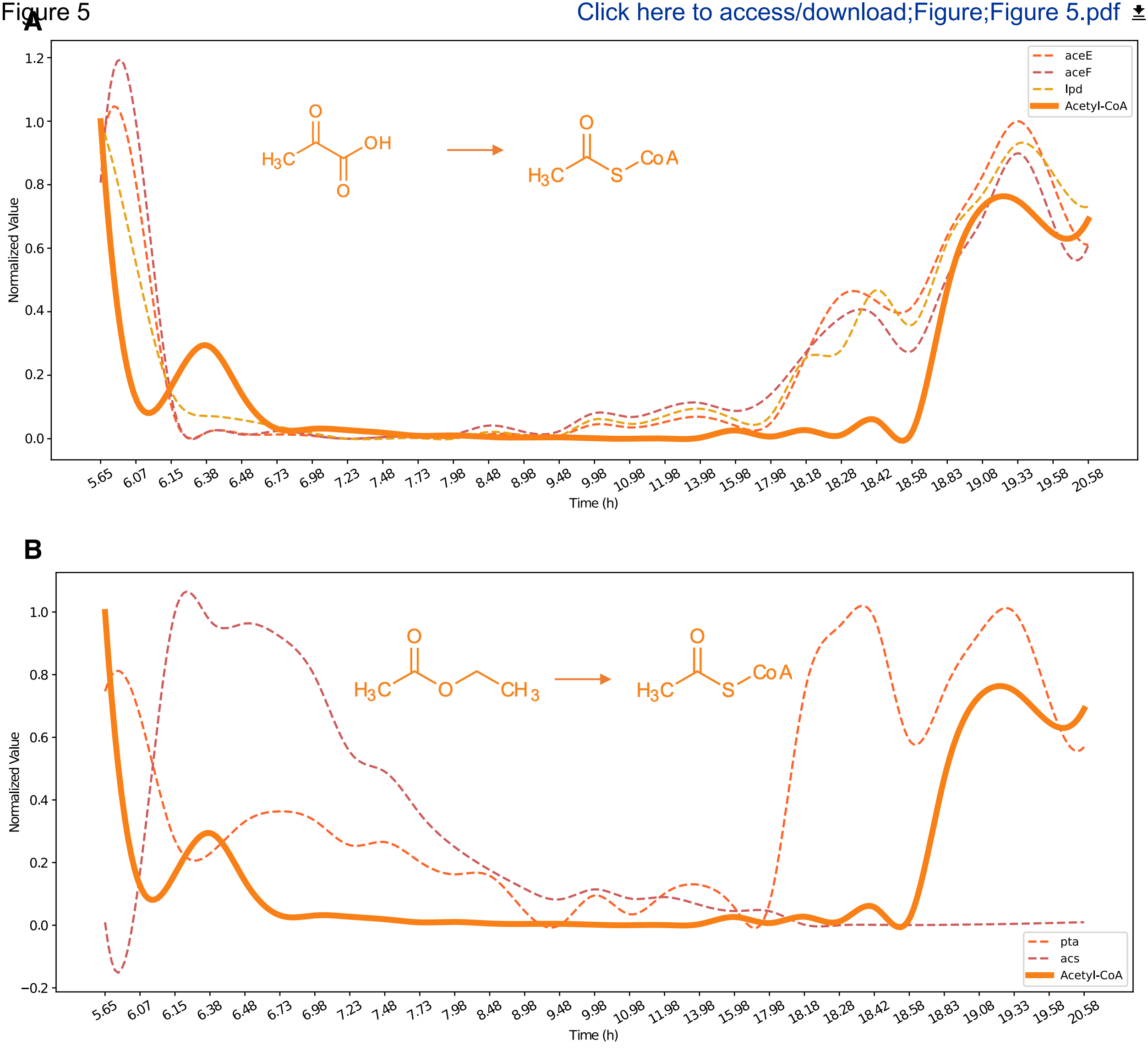

Supplementary Text

Click here to access/download
Supplementary Material
Supplementary Material for CAT Bridge (1).docx

Supplementary Figure 1

Click here to access/download
Supplementary Material
Figure S1.pdf

Supplementary Figure 2

Click here to access/download
Supplementary Material
Figure S2.pdf

Supplementary Figure 3

Click here to access/download
Supplementary Material
Figure S3.pdf

Supplementary Figure 4

Click here to access/download
Supplementary Material
Figure S4.pdf

Supplementary Table 1

Click here to access/download
Supplementary Material
Supplementary Table S2.xlsx

Supplementary Table 2

Click here to access/download
Supplementary Material
Supplementary Table S1.xlsx
