# Supplementary material for: CAT Bridge: an efficient toolkit for gene–metabolite association mining from multiomics data
Source: Gigascience. 2024 Nov 8;13:giae083. doi: 10.1093/gigascience/giae083 (PMC11548955; doi:10.1093/gigascience/giae083)
Supplement: giae083_Supplemental_Files [file giae083_supplemental_files.zip › Supplementary Material for CAT Bridge (1).docx]

**Supplementary Text for**

**CAT Bridge: An Efficient Toolkit for Gene-Metabolite Association Mining from** **Multi-Omics Data**

Bowen Yang^1,2^, Tan Meng^1^, Xinrui Wang^1^, Jun Li^1^, Shuang Zhao^3^, Yingheng Wang^4^, Shu Yi^1^, Yi Zhou^1^, Yi Zhang^1^, Liang Li^2,3,*^, Li Guo^1,*^

*^1^* *Peking University Institute of Advanced Agricultural Sciences, Shandong Provincial Key Laboratory of Precision Molecular Crop Design and Breeding, Shandong Laboratory of Advanced Agricultural Sciences at Weifang, Weifang, 261325, China*

*^2^ Department of Chemistry,* *University of Alberta, Edmonton, AB T6G 2G2, Canada*

*^3^ The Metabolomics Innovation Centre,* *University of Alberta, Edmonton, AB T6G 1C9, Canada*

*^4^ Department of Computer Science,* *Cornell University, Ithaca, NY 14853, USA*

Author Email: by8@ualberta.ca, mengtan_big_data@163.com, 1120230736@mail.nankai.edu.cn, jun.li@pku-iaas.edu.cn, szhao1@ualberta.ca, ys3702289@outlook.com, yw2349@cornell.edu, zhouyi_zoe@cau.edu.cn, 2022308310509@cau.edu.cn, liang.li@ualberta.ca, li.guo@pku-iaas.edu.cn

Correspondence should be addressed to Guo Li. Tel: +86 13325308113; Email: li.guo@pku-iaas.edu.cn

*Correspondence may also be addressed to Liang Li. Tel: +1 [(780) 492-3250](tel:7804923250); Email: [liang.li@ualberta.ca](mailto:liang.li@ualberta.ca)

**Running title**: *CAT Bridge*

**Supplementary text**

**The implementation methods of computation**

In CAT Bridge, we integrated seven different computational methods for calculating similarity or causality, including Convergent Cross Mapping (CCM) and Granger Causality (Granger) for assessing cause-and-effect relationships, and utilized Canonical Correlation Analysis (CCA), Dynamic-Time-Warping (DTW), Cross-Correlation Function (CCF), Spearman Correlation Coefficient (Spearman), and Pearson Correlation Coefficient (Pearson) for correlation calculations.

All these computations rely on Python and existing Python packages. Specifically, CCM relies on the 'causal_ccm' package, while Granger and CCF utilize 'statsmodels.tsa.stattools.' For CCA, rely on 'sklearn.cross_decomposition,' 'fastdtw' is employed for DTW calculations, and 'statsmodels.tsa.stattools' is used for both Pearson and Spearman correlation calculations. For lag-indicating parameters in some functions, 1 is set as the default value, to accommodate the lag relationship between metabolites and genes in the majority of experimental designs.

**min-max normalization**

Due to the results of different computational methods have different result value range, we utilize the MinMaxScaler function from sklearn.preprocessing to perform the following transformation, ensuring that the minimum value is set to 0 and the maximum value is set to 1:

$$x_{\text{norm}}=\frac{x-x_{\text{min}}}{x_{\text{max}}-x_{\text{min}}}$$

**Visualization and other features**

Firstly, the heatmap is used to present the abundance levels of various genes and metabolites. Such visualization facilitates the discernment of inherent patterns and prevailing trends across the dataset. Secondly, principal component analysis (PCA) is used for reducing dimensions to demonstrate the consistency of biological replicates in single-omics and multi-omics scenarios. This approach is also beneficial in multi-omics integration, ensuring that a matrix with a larger number of features (typically it is transcriptomic data), does not overshadow the combined matrix. Thirdly, the software generates variable importance in projection (VIP) plots for both metabolites and genes, highlighting features that significantly influence the data's variability. Moreover, correlation networks are designed to identify metabolites that show concentration patterns similar to the target metabolite. Finally, the platform also deploys volcano plots for displaying statistical significance against fold change for each gene between peak and decline point. For gene clustering, inspired by Mfuzz [1], the fuzzy c-means algorithm was adopted. The primary aim of this approach was to categorize genes based on expression profiles, thereby deeper insights into their interrelated functions and possible regulatory interplays.

**Software development**

We developed the CAT Bridge Python library first using Python (version 3.8.10) and R (version 3.6.3). Subsequently, we imported this library to construct the backend of both standalone applications and a web server. For standalone applications, we employed Tkinter to create the graphical user interface. As for the web server, we utilized the Django framework (version 4.2.3), with HTML/CSS and JavaScript for the front end. The entire system operates on a server running Ubuntu 20.04 LTS. The artificial intelligence (AI) agent is operationalized through the utilization of GPT 3.5 Turbo via the OpenAI API. Due to network and cybersecurity issues, the AI agent is only available in the Python Package version, and users must have an API Key to gain access.

We demonstrate how CAT Bridge usage through two case studies, and provide a tutorial to help users interpret the results.

**Artificial intelligence agent**

The artificial intelligence (AI) Agent was developed utilizing the OpenAI API to integrate large language model (LLM) GPT-3.5 turbo. The prompts of the AI agent are constructed based on the top 100 genes ranked from heuristic ranking, combining the genes and their functional annotations to create a list of formatted strings, followed by a question regarding their potential involvement in the synthesis of the target metabolite (Which one may be involved in the synthesis of [target metabolite]). To mitigate the risk of hallucinations or factual inaccuracies in the LLM's response, the ‘system' role message contextualizes the AI as a knowledgeable biological chemist.  Additionally, the temperature setting was lowered to 0.2, which resulted in more cautious and deterministic outputs [2-4]. This approach minimizes the chances of deviating from factual data, favoring factual accuracy over creative but potentially speculative responses.

**Reference**

[1] Kumar L, M EF. Mfuzz: a software package for soft clustering of microarray data. Bioinformation 2007;2:5-7.

[2] Miotto M, Rossberg N and Kleinberg B. Who is GPT-3? An exploration of personality, values and demographics. arXiv preprint arXiv:220914338. 2022.

[3] Rosoł M, Gąsior JS, Łaba J, Korzeniewski K and Młyńczak M. Evaluation of the performance of GPT-3.5 and GPT-4 on the Polish Medical Final Examination. Scientific Reports. 2023;13 1:20512. doi:10.1038/s41598-023-46995-z.

[4] Khoury J, et al. Capabilities of GPT-4 in ophthalmology: an analysis of model entropy and progress towards human-level medical question answering. British Journal of Ophthalmology. 2023:bjo-2023-324438. doi:10.1136/bjo-2023-324438.
