# Supplementary material for: CAT Bridge: an efficient toolkit for gene–metabolite association mining from multiomics data
Source: Gigascience. 2024 Nov 8;13:giae083. doi: 10.1093/gigascience/giae083 (PMC11548955; doi:10.1093/gigascience/giae083)
Supplement: giae083_Response_to_Reviewer_Comments_Original_Submission [file giae083_response_to_reviewer_comments_original_submission.pdf]

Author's Response To Reviewer Comments

Dear Editor,

Enclosed is our revised manuscript entitled “CAT Bridge: An Efficient Toolkit for Gene-Metabolite Association Mining from Multi-Omics Data” that we would like to submit again as a Technical Note to GigaScience for consideration.

We are very grateful to you and the reviewers for giving us to valuable comments and suggestions to help improve our manuscript. We have been working on revising the manuscript carefully according to these comments and questions. Particularly we have addressed the reviewer concern about the LLM-based functionality and your requests on the RRID identifiers, which are described in our point-to-point response to all comments along with the revised manuscript. We trust that the revisions have substantially improved the manuscript, making it more suitable for publication in GigaScience.

Please let me know if further information is needed regarding the submission. We are looking forward to hearing from you.

Yours sincerely,

Li Guo, Ph.D.
Principal Investigator
Peking University Institute of Advanced Agricultural Sciences
Weifang, Shandong, China
li.guo@pku-iaas.edu.cn

Response to editor comments:
I'd like to emphasize that I completely share the major concerns of reviewer #1 regarding the use of an LLM for interpreting the data. The validity and factual correctness of this approach needs to be backed up with hard evidence, and limitations and caveats properly addressed. If a convincing validation of the LLM-based functionality is not possible, it might be preferable to omit or replace the GTP integration.
Author response: Thank you for your suggestion. We have thoroughly discussed this issue in our response to Reviewer #1. We have added a description of the method and on how to reduce AI hallucination problems by using appropriate prompt words and a low-temperature setting; we have also included a warning about the risks of using this feature. If the responses generated by the Large Language Model (LLM) are still considered misleading, we are prepared to completely remove this feature from CAT Bridge until the techniques are more mature.

In addition, please register any new software application in the bio.tools and SciCrunch.org databases to receive RRID (Research Resource Identification Initiative ID) and biotoolsID identifiers, and include these in your manuscript.
Author response: We have submitted CAT Bridge to receive the Research Resource Identification Initiative ID (SCR_025410) and biotoolsID (cat_bridge). And this information has been included in the Availability of source code and requirements section of our manuscript.

Response to reviewer comments:
Reviewer #1:
The authors introduce a useful tool (CAT Bridge) for integrating multiple causal and correlative analyses for multi-omics integration, which also includes a visualization and LLM component. The authors further provide two case studies (human and plant) illustrating the utility of CAT Bridge. I believe that this work should be published, as it contributes to the field of multi-omics analysis.
Author response: We are truly grateful for your positive acknowledgment of our work on the CAT Bridge tool. We believe CAT Bridge will serve as a beneficial resource for researchers in their analytical endeavors.
However, I am very concerned about the lack of description regarding the LLM. As explained by Mittelstadt et al (https://www.nature.com/articles/s41562-023-01744-0), LLMs do not always provide factual

answers. The authors need to justify the use of the LLM to determine the relevance of a gene-metabolite association. In particular, the authors should add to the main text (or at least the supplementary) a detailed description of the prompt construction and should justify why this prompt is expected to result in factual information. Furthermore, the authors should discuss the caveats of using LLMs in this context, starting with the linked article above. I believe that the manuscript will only be publishable once this concern is addressed.
Author response: We are grateful for the reference provided, Mittelstadt et al. have thoroughly discussed the issue of LLMs regarding their potential to generate misleading information in the reference, and we acknowledge that current LLMs might not be absolutely trustworthy. However, we believe that the integration of an AI Agent is a valuable feature with acceptable limitations, especially for computational biologists who may lack domain knowledge of the molecular mechanisms underlying specific biological questions. Researchers can therefore prioritize the genes suggested by the LLM for downstream research and validation. As an additional layer of information to inspire further investigation, LLMs are not meant to replace empirical validation but to provide a starting point for experimental validation based on the synthesis of prior knowledge. Users of CAT Bridge can decide if they would like assistance from the AI agent, or trust their interpretation of the output from CAT Bridge.
To minimize the potential misguidance caused by hallucinations in the Master of Laws, we have set reasonable context settings and prompts, as well as a more conservative temperature setting (which will be elaborated on in the following content). We have also added additional disclaimers to the responses generated by the artificial intelligence, these will inform researchers that the results from the AI Agent are derived from an LLM's output, which does not ensure factual accuracy, and that manual verification is essential. Furthermore, this feature requires the user to obtain an OpenAI API key which is not provided in the web server version. We trust that users who engage with this feature generally possess a robust understanding of the operational mechanisms and inherent characteristics of LLMs' outputs.
In addition, we have added a new section detailing the LLM's methodology to the revised supplementary text: “The artificial intelligence (AI) Agent was developed utilizing the OpenAI API to integrate large language model (LLM) GPT-3.5 turbo. The prompts of the AI agent are constructed based on the top 100 genes ranked from heuristic ranking, combining the genes and their functional annotations to create a list of formatted strings, followed by a question regarding their potential involvement in the synthesis of the target metabolite (Which one may be involved in the synthesis of [target metabolite]). To mitigate the risk of hallucinations or factual inaccuracies in the LLM's response, the ‘system' role message contextualizes the AI as a knowledgeable biological chemist. Additionally, the temperature setting was lowered to 0.2, which resulted in more cautious and deterministic outputs. This approach minimizes the chances of deviating from factual data, favoring factual accuracy over creative but potentially speculative responses” (Lines 74-84).
We also incorporated a discourse on the caveats of utilizing LLMs into the Discussion: “LLM-based AI agents have shown a wide range of applications in various fields, but the hallucination of knowledge deficiency remains an issue that can mislead users. Although we have enhanced the credibility of the AI agent response via appropriate prompt and low temperature, this cannot be regarded as a substitute for professional knowledge and experimental verification, but merely serves as a starting point for verification, the definitive conclusions still require manual assessment by researchers” (Lines 387-392).
Finally, we also added this statement to the end of the AI agent response: “This response is generated by a Large Language Model (GPT 3.5 turbo) and should not be considered as a substitute for professional judgment. Users are advised to verify the accuracy of the information through rigorous literature review and, where necessary, experimental confirmation.”
If the responses generated by the LLM are still regarded as misleading, we can completely remove this feature from CAT Bridge until the techniques mature.

In addition, the authors are recommended to address the following more minor concerns:
Implementation:
Your "example file" links at https://catbridge.work are broken. Please fix this.
Author response: We apologize for the inconvenience caused by the broken links, we have addressed the issue, and the links are now functional.

Abstract:
1.Line 32: "Nevertheless, substantial challenges remain in determining the association between gene-metabolite pairs due to the complexity of cellular networks." This is not a clear statement. What about the

complexity of cellular networks presents challenges in determining the associations?
Author response: We appreciate the opportunity to clarify the statement regarding the challenges presented by the complexity of cellular networks in determining gene-metabolite associations. The complexity arises due to several factors:
●Non-linear Relationships: Gene expression and metabolite production often exhibit non-linear dynamics, complex regulatory networks, and feedback loops, making it difficult to predict based on simple correlation-based models.
●Non-one-to-one Correspondence: Multiple genes can influence a single metabolite, and conversely, a single gene can affect multiple metabolites. This complexity makes it challenging to establish direct associations.
●Temporal Dynamics: The temporal dynamics in cellular networks pose challenges, as metabolite levels often lag behind changes in gene expression. This inherent delay complicates the direct determination of their association.
Therefore, we have revised the manuscript text accordingly: "Nevertheless, substantial challenges remain in determining the association between gene-metabolite pairs due to the non-linear and multifactorial interactions within cellular networks. The complexity arises from the interplay of multiple genes and metabolites, often involving feedback loops and time-dependent regulatory mechanisms that are not easily captured by traditional analysis methods" (Lines 32-36).

2.Make sure you are using present tense consistently, not past tense (Line 39).
Author response: Thank you for the suggestion. We have revised the manuscript text to ensure consistent use of the present tense.

3.Please use the scientific name with the common name in parentheses as follows: Capsicum chinense (chili pepper). Use only the scientific name throughout the rest of the document (Line 41).
Author response: Thanks for your suggestion. We have revised the manuscript to comply with the requested format for scientific names. The species is now initially presented as Capsicum chinense (chili pepper), and only the scientific name is used throughout the remainder of the manuscript.

Background:
1.Line 56: "Background" should not be plural.
Author response: Revised.

2.Lines 59-60: More comprehensive than what? Please elaborate here.
Author response: Thanks for the questions. We meant to say that it is more comprehensive than single-omic studies. The term "comprehensive" refers to the integration of multiple layers of molecular information and inter-level regulatory relationships. This integration enables us to have a broader picture of the biological system, thus determining whether observed changes at a molecular level from broader alterations across a metabolic pathway, or only regarding a specific molecule. Therefore, it provides a more systematic understanding of the biological mechanism, making it more comprehensive than single-omic studies.
We revised the manuscript as follows: "multi-omics data analysis is crucial for understanding intricate biological mechanisms from a more comprehensive perspective than single omics, this holistic analysis allows us to explore the interplay between different molecular levels." (Lines 59-62).

3.In Line 60, please include and familiarize yourself with the following reference: Eicher, T., G. Kinnebrew, A. Patt, K. Spencer, K. Ying, Q. Ma, R. Machiraju and E. A. Mathé (2020). "Metabolomics and Multi-Omics Integration: A Survey of Computational Methods and Resources." Metabolites 10: 202.
Author response: Thank you for recommending this review paper. We have carefully reviewed the literature and cited it, as it provides a comprehensive survey of computational methods and resources for metabolomics and multi-omics integration. It specifically discusses time series studies in omics, which is the main focus of our paper. This reference emphasizes the importance of addressing the lag issues between different omics layers. It uses Dynamic Time Warping (DTW), which is also integrated into CAT Bridge, as an example to illustrate how to align time points. Such specific data types are often overlooked in the research community's reviews (Lines 62).

4.Lines 67-68: Citation needed.

Author response: We have now included the necessary citations (doi: 10.1016/j.csbj.2021.09.001) in Lines 69-70 to support our statements, ensuring that our claims are well-substantiated.

5.Line 72: Please use the scientific name with the common name in parentheses.
Author response: Thank you for the reminder, we have updated our manuscript, now listing both the scientific and common names for two species: Solanum lycopersicum (tomato) and Oryza sativa (rice) (Line 74).

6.Lines 74-77: Citations needed.
Author response: We have now included the necessary citation (doi.org/10.1039/D2NP00032F and doi:10.3390/metabo10050202) in lines 77-80 to support this statement.

7.Lines 77-78: Give an example of biologically naïve conclusions from purely data-driven strategies.
Author response: Thanks for your suggestion! To help users understand the concept, we have included a classic example in our manuscript that shows how biologically naïve conclusions came from purely data-driven strategies. The manuscript has been revised as follows: "For example, a purely data-driven approach might incorrectly link low cholesterol levels with higher mortality rates, suggesting that lower cholesterol is detrimental. However, the actual cause is underlying diseases like cancer, which cause both low cholesterol and increased mortality" (Lines 83-86).

8.Line 78: Discuss how the machine learning models could address the drawbacks of the correlation models and vice-versa.
Author response: We apologize for the ambiguity. The "two methodologies" mentioned here refers to data-driven and knowledge-driven methods. To avoid ambiguity, we revised the sentence as "Therefore, integrating data-driven and knowledge-driven methodologies may offer a more comprehensive and accurate interpretation of multi-omics data. (Lines 87-88)"
The correlation models identify new connections and provide quantitative analysis, while knowledge-driven methods avoid many false positives caused by coincidental similarities to true functional gene expression patterns in situations where data is scarce. Thus, this combination improves generalizability and increases the interpretability of the model.

Materials and Methods:
1.It seems that CAT Bridge needs to be run on one metabolite at a time. In this case, I would not use the term "gene-metabolite pair association" in Line 104, but rather "associations between genes and the target metabolite".
Author response: Thank you for your suggestion, we have made the change in our manuscript (Lines 112-113). This revision enhances the clarity of our presentation and avoids any potential misunderstanding regarding the scope of CAT Bridge.

2.Line 115: Clearly state which of these methods are non-linear and which address the lag issue.
Author response: We have provided a detailed explanation of the methods in the revised manuscript (Table 1). Convergent Cross Mapping (CCM) and Dynamic Time Warping (DTW) are non-linear methods. CCM reconstructs the state space to detect causality in non-linear systems, while DTW allows flexible matching to handle non-linear alignments of time series data. Granger Causality (Granger) and Canonical Correlation Analysis (CCA) are inherently linear methods, although there are extensions such as non-linear Granger and kernel CCA that can address non-linear relationships. For handling time lag, CCM, Granger Causality, DTW, and the Cross-Correlation Function (CCF) are capable of addressing this issue. CCM and DTW inherently consider time lag through state space reconstruction and optimal matching, respectively. Granger Causality explicitly incorporates lagged values in its regression model, and CCF measures correlation at various time lags. Although standard CCA does not handle time lags, time-lagged variations of CCA can address this problem.

3.Line 136: Your figures are out of order (Figure 1B comes after Figure 2B).
Author response: Thanks for your comment. We apologize for the mistake. We have revised the manuscript to give the correct figure reference. The previous mention of "Figure 1B" has been revised to "Figure 2B".

4.Please take a look at the Minimum Standards Reporting Checklist

(https://academic.oup.com/gigascience/pages/Minimum_Standards_of_Reporting_Checklist). In particular: a. In the section starting at Line 153, list the number of seedlings used. b. Were all timepoints collected from all seedlings? List the total number of samples. c. How many mg were collected per sample (can use a range here)? d. 3 biological replicates per seedling? Give more detail here. e. What machine was used for the ultrasonic process? If frequency settings are permitted by the machine, list the settings used. f. How many of the 28 younger and 54 older adults had both transcriptome and metabolome data?
Author response: Thanks for your suggestion, we have revised our manuscript by considering this standard:
Line 167 to 188:
“To test the effectiveness of CAT Bridge across different species, especially its applicability to non-model organisms, we collected transcriptome sequencing and metabolic profiling data from Capsicum chinense, focusing on one of its trademark natural products, capsaicin. C. chinense seedlings were divided into three groups, each containing 15 seedlings, grown in a greenhouse of Peking University Institute of Advanced Agricultural Sciences with a controlled environment of 25°C temperature, a light-dark cycle of 16 hours light and 8 hours dark, and 70% relative humidity. The fruits of C. chinense were sampled at seven distinct time points, starting from the day of flowering, i.e. 0 day post-anthesis (DPA) during which flowers were collected, followed by fruit harvest on days 7, 16, 30, 50, 55, and 60 DPA. For each time point, we sampled 15 fruits from each group of seedlings, yielding a total of 45 sampling per time point. For each group, we utilized 1.0 mL of 70% aqueous methanol per sample, with a sample weight of approximately 50mg (aside from the 7 DPA samples which averaged 27.3mg). The samples were ground and freeze-dried in liquid nitrogen. Each sample was then extracted using 1.0 mL of 70% aqueous methanol for every 50 mg of sample. Following extraction, the samples underwent ultrasonic treatment using an Ultrasonic Cell Disruptor SCIENTZ-IID (Ningbo Scientz Biotechnology Co., LTD., China) at a frequency of 40 kHz for 10 minutes at room temperature. Standards were prepared as follows: a mixed standard solution, ranging from 20-50 μg/mL, was prepared using MS-grade methanol. For the amino acid standard solution, a 1 mg/mL stock solution was initially prepared in water, and then diluted with 50% methanol to achieve a final concentration of 50 μg/mL. Three biological sample replicates were utilized in the subsequent transcriptome and metabolome analyses.”
Lines 226 - 229:
“The data was sourced from a published aging study that sampled transcriptome and metabolome data from 28 younger (20 to 25 years) and 54 older (55 to 66 years) female human donors. 13 time points are included in them where both the transcriptome and the metabolome data were detected.”

5.Line 209: "Younger" and "older" are better terms.
Author response: We appreciate your suggestion. These terms have been updated throughout the manuscript to ensure consistency and precision in age group descriptions.

Results:
1.Line 248: How does the AI agent analyze the functional annotations?
Author response: Thanks for the question. The specific description of the AI agent is discussed in our response to your previous comment above. Specifically, for this case study, the top 100 genes in heuristic ranking ranked by the CCM-based method along with their functional annotations (generated by eggNOG-mapper) were fed to a GPT 3.5-based LLM. The context was set as a professional biochemist, with the inquiry focusing on which gene might be involved in the synthesis of capsaicin. The AI agent successfully located BC332_05016, a gene encoding Acyl-transferase, a key enzyme of capsaicin biosynthesis.

2.Lines 281-282: "This illustrates the advantage of causal relationship modeling methods over traditional methods".
Author response: Thanks for your correction, we have revised the manuscript accordingly (Line 308).

3.Line 290: Please also include the updated IntLIM paper (IntLIM 2.0): Eicher, T., K. D. Spencer, J. K. Siddiqui, R. Machiraju and E. A. Mathe (2023). "IntLIM 2.0: identifying multi-omic relationships dependent on discrete or continuous phenotypic measurements." Bioinformatics Advances 3(1): vbad009.
Author response: Thank you for your suggestion on the citation. We have included this IntLIM2.0 paper to the revised manuscript (Line 339).

4.Make sure the colors are consistent in Table 1.

Author response: We have revised the table to ensure that the colors used are consistent.

5.Line 156: The scientific name of the pepper species is inconsistent with other areas of the text.
Author response: Thank you for your reminder, we have corrected the scientific name.

Figures:
1.S1 should be provided as a table, not a figure.
Author response: Thanks for your suggestion! This figure was used to show the visual output of CAT Bridge and contained content already shown in the supplementary table. Thus, we have removed Figure S1.

2.Please make S2 larger. It is difficult to read.

Author response: Revised as suggested.

3.S3 needs labels (x axis, y axis, legend).
Author response: Revised as suggested.
Thank you once again for your meticulous review of our work, especially for the detailed discussion regarding the LLMs and your attention to the scientific details within our manuscript, ensuring that it is presented with clarity, accuracy, and integrity.

Reviewer #2:
To the authors,
Thank you for the opportunity to review the manuscript GIGA-D-24-00083. The authors created a tool to predict association between genes and metabolites using various algorithms. The authors provide the tool as a web application, and as a python package. To get the reciprocal relationship between gene and metabolites, i.e. which metabolites can change which gene or vice versa, this tool can be a toolkit for the biologist or bioinformatician.
The tool has application specially the relationship between changes in genes and metabolites is not direct, many complex mechanisms exist e.g. epigenetic or polymorphism. So the tool can be alternate to other available tools.
Also, the manuscript brings the community focus on causal relationships instead of just correlation based relationships. The tool used temporal causality algorithms for predicting relationships between genes and metabolites.
Author response: Thank you for your positive comments, highlighting temporal causality between omics data and providing democratized analytical software were key objectives of our work.

However, I recommend major revisions before publication. Here are my reasons and comments for the revisions:
General issues with web accessibility and package installation :
1.There are concerns about web accessibility, as indicated by web browsers flagging the connection as insecure. This may stem from geographical restrictions or the absence of HTTPS certification. Addressing these issues would ensure secure access to the server.
Author response: Thanks for your excellent suggestion and for bringing up this issue. Currently, more and more browsers enforce stringent security protocols. Therefore, we have transitioned to using HTTPS to ensure a more secure protocol for accessing the server.

2.Despite successful initiation of the client application from the git repository as a python module, no results were generated upon launching. It is suggested that the authors distribute the tool as a Docker image to facilitate seamless usage, eliminating concerns regarding dependencies and version compatibility.
Author response: Thank you for the feedback and suggestion regarding the client application. We have created and distributed a Docker image, and it's currently accessible on Docker Hub (https://hub.docker.com/r/bowen172/cat-bridge). Additionally, we have provided complete environment information (https://github.com/Bowen999/CAT-Bridge/tree/main/client), enabling users to recreate the same environment using conda. This method is more straightforward and can avoid issues related to hardware architecture differences. By providing two files, it can avoid issues with version compatibility and environment setup.

Other comments:
1.There are inconsistencies regarding data preprocessing. While the manuscript mentions that the tool will handle preprocessing, it also indicates that users need to provide processed files. Clarification is needed on whether preprocessing is required. It seems, the tool required preprocessed data.
Author response: Thanks for your comment. The software indeed requires preprocessed gene expression matrices and metabolite concentration matrices, instead of raw data. The preprocessing mentioned in the manuscript refers to handling missing values, standardization, and other such adjustments within these matrices. We have revised the manuscript to replace preprocessing" with "data processing" for clarity.

2.For clarity use "causality and correlation" instead of "causality/correlation" algorithms.
Author response: We agree with you that this is a clearer terminology and avoids any ambiguity that might arise from the use of a slash. We have updated the manuscript to reflect this change. Thank you for the suggestion.

3.Can the tool process any new temporal numerical data series, or does it specifically filter for genes? For instance, if I provide a list of proteins along with a list of genes, will I receive the association between them? It is suggested to include this in the discussion section.
Author response: We appreciate your insightful question regarding the tool's capacity to process various temporal numerical data series.
Our paper primarily focuses on the discovery of associations between genes and metabolites. However, the algorithms integrated into CAT Bridge were originally applied in other fields. For example, Convergent Cross Mapping (CCM), the highest-performing algorithm in this study, was initially used in the ecological study. Similarly, the Granger causality test originated in an economics study. Theoretically, these algorithms have the potential to analyze time-series data of other molecular types, such as the associations between genes and proteins, proteins, and metabolites (and vice versa). To demonstrate the tool's versatility beyond gene-metabolite pairs, we have included a case study (Case Study 3) that investigates the relationship between metabolites and genes (more particularly, transcription factors). For further information, see our response to question 9 in this letter.
We have also added the following content to the Discussion regarding this issue: “We tested the performance of CAT Bridge on the gene-to-metabolite and metabolite-to-gene tasks in this research, since CAT Bridge is derived by integrating algorithms originating from different fields and its theoretical basis suggests it has generalizability, we believe that it can be used for investigating associations between other different molecular levels (such as gene to protein). Nonetheless, validating its performance across these different interactions is necessary for the conclusion due to the variations in complexity and regulatory timing at different molecular levels (Lines 380-384).”

4.Does the tool offer the capability to generate a causal diagram or network from these vectors, thereby providing visual support for their assertion regarding the causal relationship between metabolites and genes? If the author is working in this direction, it is suggested that information can be added in the discussion section.
Author response: Thanks for your question. Yes, we are actively exploring methods to visualize biological causal networks, including illustrating the time lag between metabolite concentrations and gene expression patterns, and integrating KEGG metabolic pathways information to construct molecular networks. We plan to include this functionality in our future work.
We have added the following statement to the Discussion: "Future work will involve determining response times between molecules at different levels, visualizing these lagged non-linear relationships, and constructing molecular networks based on temporal causality and metabolic pathways (Lines 400-402).

5.What definition of causal relationship did the author use, and could they provide a citation for their definition. Predictability or any other criteria were used for causal relationships. Please include the definition or criteria in the introduction and method section.
Author response: Thanks for your suggestion. The definition of causality utilized in this manuscript aligns with the concept of predictability, whereby causality is inferred based on the ability of one time series (gene expression) to predict another (metabolite concentration). This inference is implemented through the application of Convergent Cross Mapping and Granger Causality test.
To improve clarity, additional information was incorporated into the Background section with citations: "A more reliable solution is to use causality, which is inferred based on the ability of one time series (e.g.,

gene expression) to predict another (e.g., metabolite concentration), to replace the correlation coefficient (Lines 80-82)."
Similarly, the Methods section has been revised to include detailed descriptions: "including Granger Causality (Granger) and Convergent Cross Mapping (CCM) by evaluating the predictability of metabolite concentration from gene expression to represent causality (Lines 126-127)."

6.What are the minimum or maximum time points (interval) for input files? e.g. will the tool work if I provide only two times points or If I provide 48 times points. Please include the information in the method section.
Author response: Good question! In general, an increased number of sampling time points contributes to greater reliability of the results, as this mitigates the issue of false positive candidate genes that exhibit similar expression patterns to truly functional genes by coincidence. And in cases where time points are numerous, particularly with intensive sampling at short intervals, it is crucial to select a reasonable time lag. The example involving E. coli, as discussed in response to Question 9, effectively demonstrates this point.

7.What is the influence of the number of time points on the vector relationship presented in the paper? Have any studies by the authors addressed this question? Please include the results and discussion.
Author response: In our study, the influence of the number of time points primarily arises from the sampling interval, which affects the lag time settings, and whether the number of time points is sufficient to achieve comprehensive causal modeling results. The detail about this topic is discussed in Question 6 and Question 9.

8.Could the authors clarify which heuristic algorithm was employed for ranking the genes? Additionally, can they elaborate on how their approach to gene ranking is heuristic rather than relying on mathematical optimization or algorithmic methods? Clarification on the term "heuristic" would be beneficial.
Author response: The term "heuristic" refers to problem-solving techniques that employ practical methods to find solutions when classic methods are impractical. In our case, we have utilized a heuristic approach to rank genes based on their association with metabolites, rather than employing strict mathematical methods. The heuristic algorithm we employed for gene ranking integrates multiple factors, including the strength of the association between genes and metabolites as measured by various statistical methods, fold change values, and, optionally, gene function annotations. This composite scoring system is designed to prioritize genes that are more likely to be biologically relevant with the target gene. This ranking is not the result of a single, universally optimal solution but rather a practical method to prioritize candidate genes for further investigation, this approach is flexible and can be adapted based on the availability of data and specific research questions.
We did not adopt strict mathematical methods but rather heuristic ranking for the following reasons:
●Complexity of Biological Systems: Biological systems are inherently complex, with many variables and interactions that are not fully captured by strict mathematical models, especially for models applicable to different species. Heuristic methods allow for flexibility in handling this complexity.
●Data Characteristics: The data from omics studies can be noisy and high-dimensional, often with missing values or incomplete information. Heuristic approaches can be more robust to these data characteristics compared to strict mathematical methods that may require complete and clean data.
●Scalability and Adaptability: Heuristic approaches are generally more adaptable to different research questions and can be scaled to various sizes of datasets without significant modifications.

9.Could the authors offer an example from studies conducted on yeast, E. coli, or other simple organisms, demonstrating how changes in gene sequences have readily been observed to affect metabolite levels? Please include that in the results section.
Author response: Thank you for the suggestion. Yes, in simple organisms, the regulation relationship between genes and metabolites also exhibits time lag and non-linear characteristics. Previous researchers have observed these phenomena in E. coli networks (https://doi.org/10.1089%2Fomi.2010.0074). To validate this and test CAT Bridge's performance on this task, we utilized an open-sourced transcriptomics and metabolomics dataset from E. coli (https://doi.org/10.1038/s41467-019-12474-1) to perform a new case study, which we have included as a new results section in the manuscript.
We have updated the Materials and Methods and Results section of our manuscript to include the following example:

Lines 232 to 237:
"The dataset for E. coli originated from a study on the interactions between metabolites and genes within the bacterium [33]. Researchers manipulated the culture conditions of E. coli, alternating between growth and starvation phases. They then collected the transcriptome and metabolome under these varying conditions to systematically explore how metabolites influence transcription factors. The dataset includes 29 time points where both transcriptomic and metabolomic information were concurrently obtained."
Lines 311 to 331:
"In the third study, we utilized a transcriptomic and metabolomic dataset from E. coli to demonstrate the capabilities of CAT Bridge in analyzing simple organisms. The dataset contains gene expression and metabolite concentration data across 29 time points, and it was originally generated by Lempp et al. to investigate the allosteric regulation of transcription factors (TFs) by metabolites. They constructed a "literature network" of known metabolite-TF interactions, including 16 interactions as activations of the TF by metabolites. Using Kinetic Correlation, which accommodates time lags, Lempp et al. recovered 10 out of the 16 activation interactions. We re-analyzed this dataset using CAT Bridge to test its performance with simple organisms. Utilizing the functions integrated within CAT Bridge and setting a time lag of 4, we calculated the association strengths between these molecules. The CCM method once again performed best, reproducing 14 out of 16 interactions.
Using CAT Bridge, we set a time lag of 3 to investigate the genes associated with Acetyl-CoA as the target metabolite. The CCM-based methods fared the best, and the results showed strong causality (greater than 0.9) with the aceE, aceF, and lpd genes, which are involved in the conversion of pyruvate to Acetyl-CoA (Figure 5A). These genes were also ranked in the top 100 in heuristic sorting and clustered together. Additionally, the acs and pta genes, which are involved in the conversion of acetate to Acetyl-CoA, also showed strong causality (0.840 and 0.721, respectively) but did not rank in the top 100 in heuristic sorting (Figure 5B). This suggests that in this study, the precursors for Acetyl-CoA may predominantly come from pyruvate."
We have also updated the Materials and Methods section (Lines 232-237): "The dataset for E. coli originated from a study on the interactions between metabolites and genes within the bacterium [33]. Researchers manipulated the culture conditions of E. coli, alternating between growth and starvation phases. They then collected the transcriptome and metabolome under these varying conditions to systematically explore how metabolites influence transcription factors. The dataset includes 29 time points where both transcriptomic and metabolomic information were concurrently obtained".

10.Does the tool generate a vector indicating many-to-many relationships or one-to-one relationships? In other words, does it reveal whether one gene is associated with many metabolites, and vice versa, or if it establishes a single gene-metabolite relationship? Please include this in the results section. Also, in the discussion section please include examples of application of these relationships in various fields e.g. metabolic engineering or cancer metabolism.
Author response: Thanks for your question. CAT Bridge generates a vector for each gene in relation to a given target metabolite. Therefore, for each run, many vectors will be generated, and each vector represents a single gene-to-metabolite relationship.
We have revised the Methods section to include this clarification: "The algorithm chosen by the user will then generate a vector for each gene representing one-to-one associations between the gene and the target metabolite (Lines 111-113)."
We also updated the Discussion section: "We believe that discovering associated gene-metabolite pairs will have practical applications in many fields. For example, in metabolic engineering, a single metabolite is often regulated by multiple genes, by comparing the association strength, one can infer which genes may play a dominant role under certain conditions, facilitating the development of targeted metabolic engineering strategies. Furthermore, in horticulture and breeding, identifying genes with causal relationships to key natural products, particularly in non-model organisms, can significantly aid in molecular breeding efforts, potentially leading to the development of new crop varieties with enhanced yields or desired characteristics (Lines 363-371)."

11.Table 1 compares the features of CAT Bridge with other available methods. It should encompass features provided by other tools that are not available in the author's tool, such as knowledge-driven integration or integration with a third-party database. Additionally, it should address the limitation posed by the requirement of time series data, which is not just a strength but also a challenge, particularly for epidemiology studies where multiple time series for gene expression may not be feasible.

Author response: Thanks for your suggestion. We have revised the Table 2 to provide a more comprehensive comparison of CAT Bridge with other available tools in the field to include a broader range of features. We highlighted not only the strengths of CAT Bridge but also notable features of other tools that are not present in CAT Bridge, include third-party database connectivity, knowledge-driven analysis, and integration.
On the other hand, we would like to emphasize that CAT Bridge is currently designed to provide a solution for longitudinal multi-omics studies, rather than attempting to offer a universal solution for all multi-omics research. For fields where sampling across multiple time points is challenging, such as epidemiology, we recommend the use of established methods like Mendelian Randomization or structural equation modeling for causal inference or checking conformity with the Bradford Hill criteria.

12.Please use alternative phrases to "Self-generated data," such as "experimentally obtained data," to clarify that the author is utilizing data acquired in the lab to validate the tool. (e.g. line 42, 223, and 492).
Author response: Per your suggestion, we have revised the manuscript to replace the term "self-generated data" with "experimentally obtained data" to clarify that the data were acquired through laboratory experiments. This change has been applied to the specified lines and throughout the document where necessary (Lines 44, 251, 606, and Figure 1).
We would like to express our gratitude to both reviewers again for your meticulous review and insightful comments that help improve our manuscript.
