# Supplementary material for: CAT Bridge: an efficient toolkit for gene–metabolite association mining from multiomics data
Source: Gigascience. 2024 Nov 8;13:giae083. doi: 10.1093/gigascience/giae083 (PMC11548955; doi:10.1093/gigascience/giae083)
Supplement: giae083_Response_to_Reviewer_Comments_Revision_1 [file giae083_response_to_reviewer_comments_revision_1.pdf]

Author's Response To Reviewer Comments

Dear Hans,

We have made the requested changes to the manuscript according to your comments. The manuscript file is uploaded for your review. Please let me know if you have further questions.

Thank you for considering our work for publication in GigaScience.

Sincerely,
Li Guo
