# Supplementary material for: CAT Bridge: an efficient toolkit for gene–metabolite association mining from multiomics data
Source: Gigascience. 2024 Nov 8;13:giae083. doi: 10.1093/gigascience/giae083 (PMC11548955; doi:10.1093/gigascience/giae083)
Supplement: giae083_Reviewer_1_Report_Original_Submission — Tara Eicher -- 4/18/2024 Reviewed [file giae083_reviewer_1_report_original_submission.pdf]

Reviewer Report

Title: CAT Bridge: An Efficient Toolkit for Gene-Metabolite Association Mining from Multi-Omics Data

Version: Original Submission **Date:** 4/18/2024

Reviewer name: Tara Eicher

Reviewer Comments to Author:

The authors introduce a useful tool (CAT Bridge) for integrating multiple causal and correlative analyses for multi-omics integration, which also includes a visualization and LLM component. The authors further provide two case studies (human and plant) illustrating the utility of CAT Bridge. I believe that this work should be published, as it contributes to the field of multi-omics analysis.However, I am very concerned about the lack of description regarding the LLM. As explained by Mittelstadt et al (https://www.nature.com/articles/s41562-023-01744-0), LLMs do not always provide factual answers. The authors need to justify the use of the LLM to determine the relevance of a gene-metabolite association. In particular, the authors should add to the main text (or at least the supplementary) a detailed description of the prompt construction and should justify why this prompt is expected to result in factual information. Furthermore, the authors should discuss the caveats of using LLMs in this context, starting with the linked article above. I believe that the manuscript will only be publishable once this concern is addressed.In addition, the authors are recommended to address the following more minor concerns:Implementation:1. Your "example file" links at https://catbridge.work are broken. Please fix this.Abstract:1. Line 32: "Nevertheless, substantial challenges remain in determining the association between gene-metabolite pairs due to the complexity of cellular networks." This is not a clear statement. What about the complexity of cellular networks presents challenges in determining the associations?2. Make sure you are using present tense consistently, not past tense (Line 39).3. Please use the scientific name with the common name in parentheses as follows: Capsicum chinense (chili pepper). Use only the scientific name throughout the rest of the document (Line 41).Background:1. Line 56: "Background" should not be plural.2. Lines 59-60: More comprehensive than what? Please elaborate here.3. In Line 60, please include and familiarize yourself with the following reference: Eicher, T., G. Kinnebrew, A. Patt, K. Spencer, K. Ying, Q. Ma, R. Machiraju and E. A. MathÃ© (2020). "Metabolomics and Multi-Omics Integration: A Survey of Computational Methods and Resources." Metabolites 10: 202.4. Lines 67-68: Citation needed.5. Line 72: Please use the scientific name with the common name in parentheses.6. Lines 74-77: Citations needed.7. Lines 77-78: Give an example of biologically naÃ¯ve conclusions from purely data-driven strategies.8. Line 78: Discuss how the machine learning models could address the drawbacks of the correlation models and vice-versa.Materials and Methods:1. It seems that CAT Bridge needs to be run on one metabolite at a time. In this case, I would not use the term "gene-metabolite pair association" in Line 104, but rather "associations between genes and the target metabolite".2. Line 115: Clearly state which of these methods are non-linear and which address the lag issue.3. Line 136: Your figures are out of order (Figure 1B comes after Figure 2B).4. Please take a look at the Minimum Standards Reporting Checklist (https://academic.oup.com/gigascience/pages/Minimum_Standards_of_Reporting_Checklist). In

particular:a. In the section starting at Line 153, list the number of seedlings used.b. Were all timepoints collected from all seedlings? List the total number of samples.c. How many mg were collected per sample (can use a range here)?d. 3 biological replicates per seedling? Give more detail here.e. What machine was used for the ultrasonic process? If frequency settings are permitted by the machine, list the settings used.f. How many of the 28 younger and 54 older adults had both transcriptome and metabolome data?5. Line 209: "Younger" and "older" are better terms.Results:1. Line 248: How does the AI agent analyze the functional annotations?2. Lines 281-282: "This illustrates the advantage of causal relationship modeling methods over traditional methods".3. Line 290: Please also include the updated IntLIM paper (IntLIM 2.0): Eicher, T., K. D. Spencer, J. K. Siddiqui, R. Machiraju and E. A. Mathe (2023). "IntLIM 2.0: identifying multi-omic relationships dependent on discrete or continuous phenotypic measurements." Bioinformatics Advances 3(1): vbad009.4. Make sure the colors are consistent in Table 1.5. Line 156: The scientific name of the pepper species is inconsistent with other areas of the text.Figures:1. S1 should be provided as a table, not a figure.2. Please make S2 larger. It is difficult to read.3. S3 needs labels (x axis, y axis, legend).

Level of Interest

Please indicate how interesting you found the manuscript: Choose an item.

Quality of Written English

Please indicate the quality of language in the manuscript: Choose an item.

Declaration of Competing Interests

Please complete a declaration of competing interests, considering the following questions:

- Have you in the past five years received reimbursements, fees, funding, or salary from an organisation that may in any way gain or lose financially from the publication of this manuscript, either now or in the future?
- Do you hold any stocks or shares in an organisation that may in any way gain or lose financially from the publication of this manuscript, either now or in the future?
- Do you hold or are you currently applying for any patents relating to the content of the manuscript?
- Have you received reimbursements, fees, funding, or salary from an organization that holds or has applied for patents relating to the content of the manuscript?
- Do you have any other financial competing interests?
- Do you have any non-financial competing interests in relation to this paper?

If you can answer no to all of the above, write 'I declare that I have no competing interests' below. If your reply is yes to any, please give details below.

I declare that I have no competing interests

I agree to the open peer review policy of the journal. I understand that my name will be included on my report to the authors and, if the manuscript is accepted for publication, my named report including any attachments I upload will be posted on the website along with the authors' responses. I agree for my report to be made available under an Open Access Creative Commons CC-BY license (http://creativecommons.org/licenses/by/4.0/). I understand that any comments which I do not wish to be included in my named report can be included as confidential comments to the editors, which will not be published.

Choose an item.

To further support our reviewers, we have joined with Publons, where you can gain additional credit to further highlight your hard work (see: https://publons.com/journal/530/gigascience). On publication of this paper, your review will be automatically added to Publons, you can then choose whether or not to claim your Publons credit. I understand this statement.

Yes Choose an item.
