# Supplementary material for: CAT Bridge: an efficient toolkit for gene–metabolite association mining from multiomics data
Source: Gigascience. 2024 Nov 8;13:giae083. doi: 10.1093/gigascience/giae083 (PMC11548955; doi:10.1093/gigascience/giae083)
Supplement: giae083_Reviewer_2_Report_Original_Submission — JITENDRA KUMAR Barupal -- 5/19/2024 Reviewed [file giae083_reviewer_2_report_original_submission.pdf]

Reviewer Report

Title: CAT Bridge: An Efficient Toolkit for Gene-Metabolite Association Mining from Multi-Omics Data

Version: Original Submission **Date:** 5/19/2024

Reviewer name: JITENDRA KUMAR Barupal

Reviewer Comments to Author:

To the authors,Thank you for the opportunity to review the manuscript GIGA-D-24-00083. The authors created a tool to predict association between genes and metabolites using various algorithms. The authors provide the tool as a web application, and as a python package. To get the reciprocal relationship between gene and metabolites, i.e. which metabolites can change which gene or vice versa, this tool can be a toolkit for the biologist or bioinformatician.The tool has application specially the relationship between changes in genes and metabolites is not direct, many complex mechanisms exist e.g. epigenetic or polymorphism. So the tool can be alternate to other available tools.Also, the manuscript brings the community focus on causal relationships instead of just correlation based relationships. The tool used temporal causality algorithms for predicting relationships between genes and metabolites.However, I recommend major revisions before publication. Here are my reasons and comments for the revisions:General issues with web accessibility and package installation :1. There are concerns about web accessibility, as indicated by web browsers flagging the connection as insecure. This may stem from geographical restrictions or the absence of HTTPS certification. Addressing these issues would ensure secure access to the server.2. Despite successful initiation of the client application from the git repository as a python module, no results were generated upon launching. It is suggested that the authors distribute the tool as a Docker image to facilitate seamless usage, eliminating concerns regarding dependencies and version compatibility.Other comments :1. There are inconsistencies regarding data preprocessing. While the manuscript mentions that the tool will handle preprocessing, it also indicates that users need to provide processed files. Clarification is needed on whether preprocessing is required. It seems, the tool required preprocessed data.2. For clarity use "causality and correlation" instead of "causality/correlation" algorithms.3.Can the tool process any new temporal numerical data series, or does it specifically filter for genes? For instance, if I provide a list of proteins along with a list of genes, will I receive the association between them? It is suggested to include this in the discussion section.4.Does the tool offer the capability to generate a causal diagram or network from these vectors, thereby providing visual support for their assertion regarding the causal relationship between metabolites and genes? If the author is working in this direction, it is suggested that information can be added in the discussion section.5. What definition of causal relationship did the author use, and could they provide a citation for their definition. Predictability or any other criteria were used for causal relationships. Please include the definition or criteria in the introduction and method section.6. What are the minimum or maximum time points (interval) for input files? e.g. will the tool work if I provide only two times points or If I provide 48 times points. Please include the information in the method section.7. What is the influence of the number of time points on the vector relationship presented in the paper? Have any studies by the authors addressed this question? Please include the

results and discussion.8. Could the authors clarify which heuristic algorithm was employed for ranking the genes? Additionally, can they elaborate on how their approach to gene ranking is heuristic rather than relying on mathematical optimization or algorithmic methods? Clarification on the term "heuristic" would be beneficial.9. Could the authors offer an example from studies conducted on yeast, E. coli, or other simple organisms, demonstrating how changes in gene sequences have readily been observed to affect metabolite levels? Please include that in the results section.10. Does the tool generate a vector indicating many-to-many relationships or one-to-one relationships? In other words, does it reveal whether one gene is associated with many metabolites, and vice versa, or if it establishes a single gene-metabolite relationship? Please include this in the results section. Also, in the discussion section please include examples of application of these relationships in various fields e.g. metabolic engineering or cancer metabolism.11. Table 1 compares the features of CAT Bridge with other available methods. It should encompass features provided by other tools that are not available in the author's tool, such as knowledge-driven integration or integration with a third-party database. Additionally, it should address the limitation posed by the requirement of time series data, which is not just a strength but also a challenge, particularly for epidemiology studies where multiple time series for gene expression may not be feasible.12. Please use alternative phrases to "Self-generated data," such as "experimentally obtained data," to clarify that the author is utilizing data acquired in the lab to validate the tool. (e.g. line 42, 223, and 492).

Level of Interest

Please indicate how interesting you found the manuscript: Choose an item.

Quality of Written English

Please indicate the quality of language in the manuscript: Choose an item.

Declaration of Competing Interests

Please complete a declaration of competing interests, considering the following questions:

- Have you in the past five years received reimbursements, fees, funding, or salary from an organisation that may in any way gain or lose financially from the publication of this manuscript, either now or in the future?
- Do you hold any stocks or shares in an organisation that may in any way gain or lose financially from the publication of this manuscript, either now or in the future?
- Do you hold or are you currently applying for any patents relating to the content of the manuscript?
- Have you received reimbursements, fees, funding, or salary from an organization that holds or has applied for patents relating to the content of the manuscript?
- Do you have any other financial competing interests?
- Do you have any non-financial competing interests in relation to this paper?

If you can answer no to all of the above, write 'I declare that I have no competing interests' below. If your reply is yes to any, please give details below.

I declare that I have no competing interests

I agree to the open peer review policy of the journal. I understand that my name will be included on my report to the authors and, if the manuscript is accepted for publication, my named report including any attachments I upload will be posted on the website along with the authors' responses. I agree for my report to be made available under an Open Access Creative Commons CC-BY license (http://creativecommons.org/licenses/by/4.0/). I understand that any comments which I do not wish to be included in my named report can be included as confidential comments to the editors, which will not be published.

Choose an item.

To further support our reviewers, we have joined with Publons, where you can gain additional credit to further highlight your hard work (see: https://publons.com/journal/530/gigascience). On publication of this paper, your review will be automatically added to Publons, you can then choose whether or not to claim your Publons credit. I understand this statement.

Yes Choose an item.
